# Vaccination Timeliness Among US Children Aged 0-19 Months, National Immunization Survey–Child 2011-2021

**DOI:** 10.1001/jamanetworkopen.2024.6440

**Published:** 2024-04-12

**Authors:** Sophia R. Newcomer, Sarah Y. Michels, Alexandria N. Albers, Rain E. Freeman, Jon M. Graham, Christina L. Clarke, Jason M. Glanz, Matthew F. Daley

**Affiliations:** 1 Center for Population Health Research, University of Montana, Missoula; 2 School of Public and Community Health Sciences, University of Montana, Missoula; 3 College of Public Health, University of South Florida, Tampa; 4 Department of Mathematics, University of Montana, Missoula; 5 Institute for Health Research, Kaiser Permanente Colorado, Aurora; 6 Department of Epidemiology, University of Colorado School of Public Health, Aurora; 7 Department of Pediatrics, University of Colorado School of Medicine, Aurora

## Abstract

**Importance:**

Delays in receiving vaccinations lead to greater vaccine-preventable disease risk. Timeliness of receipt of recommended vaccinations is not routinely tracked in the US, either overall or for populations that have known barriers to accessing routine health care, including lower-income families and children.

**Objective:**

To measure vaccination timeliness among US children aged 0 to 19 months, overall and by socioeconomic indicators.

**Design, Setting, and Participants:**

This serial, cross-sectional study analyzed nationally representative data from the 2011 to 2021 National Immunization Survey–Child (NIS-Child), an annual survey of parents, with immunization histories collected from clinicians administering vaccines. The 2020 and 2021 surveys largely reflected vaccinations in the US before the COVID-19 pandemic. Study participants included US children surveyed at ages 19 to 35 months. Data were analyzed from January to August 2023.

**Exposure:**

Survey year.

**Main Outcomes and Measures:**

The primary outcomes were average days undervaccinated (ADU) and percentage of children who received all vaccine doses on time (ie, 0 days undervaccinated) for the combined 7-vaccine series up to age 19 months. The mean adjusted annual change in on-time vaccination by socioeconomic indicators was calculated by use of multivariable log-linked binomial regression models.

**Results:**

The surveys included 179 154 children (92 248 boys [51.2%]); 74 479 (31.4%, weighted) lived above the federal poverty level with more than $75 000 in annual family income, 58 961 (32.4%) lived at or above the poverty level with $75 000 or less in annual family income, and 39 564 (30.2%) lived below the poverty level. Overall, the median (IQR) ADU for the combined 7-vaccine series in the US decreased from 22.3 (0.4-71.5) days in the 2011 survey to 11.9 (0.0-55.5) days in the 2021 survey. The prevalence of on-time receipt of the combined 7-vaccine series increased from 22.5% (95% CI, 21.4%-23.6%) to 35.6% (95% CI, 34.2%-37.0%). Although children with more than $75 000 in annual family income had a 4.6% (95% CI, 4.0%-5.2%) mean annual increase in on-time vaccination, the mean annual increase was 2.8% (95% CI, 2.0%-3.6%) for children living at or above the poverty level with $75 000 or less in annual family income and 2.0% (95% CI, 1.0%-3.0%) for children living below the poverty level.

**Conclusions and Relevance:**

In this cross-sectional study of NIS-Child data, improvements in vaccination timeliness were observed from the 2011 to the 2021 survey. However, widening disparities by socioeconomic indicators signal that increased efforts to facilitate timely vaccination among children in lower-income families are needed.

## Key Points

**Question:**

Have there been changes in timeliness of vaccine receipt among US children aged 0 to 19 months, and did changes in on-time vaccination differ by socioeconomic indicators?

**Findings:**

In this cross-sectional study of 2011 to 2021 National Immunization Survey–Child data for 179 154 children, receipt of the combined 7-vaccine series by recommended ages increased from 22.5% in 2011 to 35.6% in 2021. However, mean annual rates of improvement in on-time vaccination were lower for children from lower-income families and for children without private health insurance.

**Meaning:**

These findings suggest that vaccination timeliness has improved overall, but widening disparities by socioeconomic status indicate continued barriers to accessing routine and timely vaccinations.

## Teaser

This cross-sectional study using data from the 2011 to 2021 National Immunization Survey–Child examines vaccination timeliness among US children aged 0 to 19 months, overall and by socioeconomic indicators.

## Introduction

The immunization schedule recommended by the US Advisory Committee on Immunization Practices (ACIP) details how vaccinations should be provided at birth and ages 2, 4, 6, 12 to 15, and 15 to 18 months. National vaccination coverage is typically measured as the percentage of US children who have received recommended vaccinations by a certain age, such as by their second birthday.^1^ However, these coverage measures do not reflect whether children received vaccinations on time, by the ages recommended by the ACIP.

There are several reasons that timeliness of vaccine receipt is important for children’s health. Delays in receiving vaccinations lead to increased risk of vaccine-preventable diseases, including measles and pertussis.^2,3,4^ Furthermore, children who fall behind are less likely to finish vaccinations that require multiple doses by the ages recommended by the ACIP.^5,6,7^ Moreover, timely receipt of preventive care is an essential component of pediatric health care quality.^8^ Surveillance of timely receipt of preventive care, including vaccinations, is particularly important for assessing progress toward eliminating disparities in children’s health.^9^ The federal Vaccines for Children program, which provides free vaccines to eligible children, led to marked improvements in vaccination coverage in the US, particularly among low-income children.^10,11,12^ However, timeliness of vaccine receipt has not been routinely tracked in the US, either overall or across socioeconomic groups.

Our primary objective was to measure vaccination timeliness for US children aged 0 to 19 months, using data from the annual National Immunization Survey–Child (NIS-Child) from 2011 through 2021. Our secondary objective was to determine whether temporal changes in on-time vaccination differed by socioeconomic indicators, including poverty and health insurance status.

## Methods

### Data Source

The University of Montana institutional review board approved this cross-sectional study under the exempt category of review. Informed consent was not sought because all data were publicly available and deidentified, in accordance with 45 CFR §46. We adhered to the Strengthening the Reporting of Observational Studies in Epidemiology (STROBE) reporting guidelines for cross-sectional studies.

The NIS-Child is a nationally representative, annual survey of parents and caregivers of children aged 19 to 35 months.^13,14,15,16^ The survey starts with a telephone interview of the parent and/or caregiver, followed by a questionnaire mailed to the child’s clinicians who administered vaccines to obtain verified vaccination records. Weights are produced through multistage procedures so that the survey sample can be representative at national, state, and some local levels.^14^

We analyzed public-use NIS-Child data files.^17^ These files included data on the age in days that surveyed children received vaccinations; however, specific dates or birth years were not provided. In 2011, the NIS-Child shifted to a dual-frame sampling design that included both landlines and cellular telephones; before 2011, only landlines were sampled.^14,18,19^ We analyzed data starting with 2011 and through the 2021 survey, the most recent year with publicly available data. Since the 2021 survey includes children born 2018 to 2020, that data set reflects vaccinations given both before and after the start of the COVID-19 pandemic. Our analyses included children from 50 US states and the District of Columbia for whom clinician-verified immunization histories were available.

### Vaccines

We focused on quantifying timeliness for vaccinations included in the combined 7-vaccine series, which is the main combined measure used by the Centers for Disease Control and Prevention for surveillance across multiple vaccinations in early childhood, and includes diphtheria-tetanus-acellular pertussis (4 doses), inactivated poliovirus (3 doses), measles-mumps-rubella (1 dose), hepatitis B (HepB; 3 doses), *Haemophilus influenzae* type b (Hib; 3 or 4 doses, depending on brand), varicella (1 dose), and pneumococcal conjugate (4 doses) vaccinations.^1^ We also calculated the timeliness of rotavirus and hepatitis A vaccinations. We did not assess the timeliness of influenza vaccinations because recommendations are for annual vaccination after age 6 months during influenza seasons and are not tied to specific age intervals.

### Days Undervaccinated

Days undervaccinated describes the number of days a child is lacking a recommended vaccination.^20^ Because ACIP recommendations for age at vaccination are provided as either 1-month or multimonth intervals, a child accrues days undervaccinated once the interval has elapsed and until the vaccine is received, or until the end of the follow-up period for children who never received the vaccine (eFigure in Supplement 1). Because all vaccinations in the combined 7-vaccine series are recommended to be received by age 19 months, our follow-up period for quantifying days undervaccinated was through age 581 days (because age-in-days variables, and not specific dates, were available in NIS-Child public-use data). Because all children were surveyed during or after 19 months of age, there was no loss to follow-up. Although children can get caught up on vaccinations later, we only analyzed vaccinations administered up until age 581 days, because our focus was quantifying timeliness relative to standard ACIP recommendations.

We used 3 information sources to establish parameters for calculating days undervaccinated. First, we reviewed annual ACIP-recommended childhood immunization schedules from 2011 to 2021 and summarized changes that may have impacted measurement of days undervaccinated.^21^ Second, we reviewed publicly available documentation from the Centers for Disease Control and Prevention’s Clinical Decision Support for Immunizations (CDSi) initiative, which supports incorporation of ACIP recommendations into clinical immunization technology.^22^ Then, we compared ACIP recommendations and CDSi documentation with parameters reported in the analysis of timeliness using 2003 NIS-Child data by Luman et al^20^ and from subsequent studies^7,23,24,25,26^ that measured days undervaccinated (eTable 1 in Supplement 1). Details on how we reconciled information from across sources to determine whether vaccinations met the minimum acceptable age of receipt and minimum acceptable interval from a prior dose of the same vaccination, and the age in days to initiate counting days undervaccinated for each vaccination, are described in the eAppendix in Supplement 1. The parameters used in our study are in Table 1.

**Table 1.** Parameters for Measuring Days Undervaccinated in Early Childhood

Vaccine and dose	ACIP-recommended age, mo	Minimum acceptable age of receipt, d^a^	Minimum acceptable interval from prior dose, d^a^	Days undervaccinated count initiation, d
Hepatitis B				
Dose 1	0	0	NA	31
Dose 2	1-2	24	24	92
Dose 3^b^	6-18	164	52	581
Doses 1-3^c^	NA	NA	108	NA
Diphtheria, tetanus, and acellular pertussis				
Dose 1	2	38	NA	92
Dose 2	4	66	24	153
Dose 3	6	94	24	215
Dose 4	15-18	361	116	581
*Haemophilus influenzae* type b				
Dose 1	2	38	NA	92
Dose 2	4	66	24	153
Dose 3^d^	6	94	24	215
Dose 4^d^	12-15	361	52	489
Pneumococcal conjugate vaccine				
Dose 1	2	38	NA	92
Dose 2	4	66	24	153
Dose 3	6	94	24	215
Dose 4	12-15	361	52	489
Inactivated poliovirus vaccine				
Dose 1	2	38	NA	92
Dose 2	4	66	24	153
Dose 3	6-18	94	24	581
Measles, mumps, and rubella, dose 1	12-15	361	NA	489
Varicella, dose 1	12-15	361	NA	489
Rotavirus^e^				
Dose 1	2	38	NA	92
Dose 2	4	66	24	153
Dose 3	6	94	24	215
Hepatitis A, dose 1	12	361	NA	550

Abbreviations: ACIP, Advisory Committee on Immunization Practices; NA, not applicable.

^a^ Allows for 4-day grace period.

^b^ When 4 doses are administered, substitute dose 4 for dose 3 in these calculations.

^c^ In 2012, the ACIP published additional guidance specifying a minimum acceptable interval between the first and third dose of hepatitis B of 16 weeks, which was calculated to be 108 days (per ACIP and Centers for Disease Control and Prevention Clinical Decision Support for Immunizations recommendations, 16 weeks × 7 days = 112 days – 4-day grace period = 108 days).

^d^ Depends on manufacturing brand. Per ACIP guidance, if *Haemophilus influenzae* type b (Hib) doses 1 and 2 were PedvaxHIB or COMVAX (both manufactured by Merck & Co, Inc), then a 6-month dose was not required. Vaccine doses of these 3-dose Hib series were considered delayed at ages 3 months (92 days), 5 months (153 days), and 16 months (489 days), respectively. For all other Hib vaccine manufacturing brands, 4 doses are needed.

^e^ The maximum age for rotavirus dose 1 is 14 weeks, 6 days. Doses received after this age were considered invalid, and days undervaccinated were not calculated beyond 15 weeks (105 days). The maximum age for the final dose is 8 months, 0 days. The number of recommended doses depends on manufacturing brand. Rotarix (GlaxoSmithKline) is a 2-dose series, and RotaTeq (Merck & Co, Inc) is a 3-dose series. If any dose is either RotaTeq or unknown, default to 3-dose series.

### Statistical Analysis

For each survey year, we reported vaccination coverage (ie, receipt of all recommended doses) by age 19 months for the combined 7-vaccine series. We then reported the median (IQR) and the mean (SE) for days undervaccinated by age 19 months for recommended vaccinations. We also reported these descriptive statistics for average days undervaccinated (ADU) for the combined 7-vaccine series, calculated as the summed number of days undervaccinated across the 7 vaccinations, divided by 7.^26^

For each vaccination individually and for the combined 7-vaccine series measure, we calculated and plotted the percentage of children who received vaccinations on time (ie, 0 days undervaccinated), by survey year. Because visual inspections showed that temporal changes were linear, we used a log-linked binomial regression model with survey year as a continuous independent variable to calculate the unadjusted mean annual change in the proportion of children vaccinated on time for the combined 7-vaccine series. We then used a multivariable log-linked binomial regression model to estimate the adjusted mean annual change in on-time receipt of the combined 7-vaccine series, controlling for poverty status, insurance status, sex, self-reported race and ethnicity, US Census region, whether the child had moved across state lines, the number of clinicians administering vaccines to the child, the types of facilities where vaccines were received, maternal education, number of children younger than 18 years in the household, and home ownership.^27^ We chose to adjust for these covariates since they may be associated with on-time vaccination and there may have been temporal changes in these variables. Race and ethnicity were included as a variable in the multivariable model because there are differences in vaccination coverage by race and ethnicity,^1^ and the distribution of race and ethnicity may have changed across survey years. Poverty status was based on US Census poverty thresholds for each survey year, defined in NIS-Child as 3 categories: above the federal poverty level and total family income greater than $75 000, at or above poverty and income $75 000 or less, or below the federal poverty level. In the 2016 to 2021 NIS-Child surveys, insurance coverage was provided as 1 variable with 4 categories: private insurance only, any Medicaid, other insurance types, and uninsured. The 2011 to 2015 surveys collected insurance data through multiple questions. For consistency, we recoded these multiple variables into 1 variable so that insurance type was categorized into the 4 categories used in the 2016 to 2021 surveys.^27^ Because it is known that vaccination coverage lags for low-income children and children without private health insurance,^1^ we also conducted a similar multivariable model limited to children who had completed the combined 7-vaccine series by age 19 months.

We then plotted the prevalence of on-time receipt of the combined 7-vaccine series by poverty status and health insurance status. We calculated the percentage point change in on-time receipt from 2011 to 2021 for each level of poverty status and health insurance. Bootstrap normal-based 95% CIs for these differences were constructed by resampling households 2000 times within each year and stratum. Using multivariable log-linked binomial regression models, we tested interaction terms to assess whether the mean annual change in on-time vaccination differed by these socioeconomic indicators. We first tested an interaction between survey year and child’s poverty status. Because the interaction was statistically significant (*P* < .05), we then fit separate models taking turns with each of the 3 poverty levels as the reference group. In each model, the adjusted prevalence ratio (aPR) for survey year represents the mean adjusted annual change in the outcome for that poverty level reference group. We repeated the same modeling processes in a separate multivariable model assessing an interaction between survey year and insurance.

All multivariable models included children with complete covariate data. Multivariable models were tested for multicollinearity, and no variance inflation was observed.^28^ Analyses accounted for the complex survey design and incorporated survey weights. Statistical tests were 2 sided, and our a priori level of significance was *P* < .05. Most analyses were conducted using SAS statistical software version 9.4 (SAS Institute); however, calculations of the bootstrapped 95% CIs were created using R statistical software version 4.1.2 (R Project for Statistical Computing). Data were analyzed from January to August 2023.

## Results

We analyzed data from 179 154 children (92 248 boys [51.2%]) with clinician-verified vaccination records in the 2011 to 2021 NIS-Child surveys; 74 479 children (weighted percentage, 31.4%) lived above the poverty level with greater than $75 000 family income, 58 961 children (32.4%) lived above the poverty level with $75 000 or less family income, 39 564 children (30.2%) lived below the poverty level, and 6150 children (6.0%) were missing poverty status data. Also, 96 284 children (43.1%) had private health insurance only, 61 461 children (44.0%) were covered by Medicaid, 9073 children (4.6%) had another form of health insurance, 8229 children (5.5%) were uninsured at any time, and 4107 children (2.9%) were missing insurance data. Although more granular self-reported race and ethnicity data are collected during the household interviews, only the following categories of race and ethnicity were available in public-use data: Hispanic (35 396 children [27.1%]), non-Hispanic Black only (14 986 children [12.9%]), non-Hispanic White only (104 908 children [46.9%]), and non-Hispanic other and multiple races (23 864 children [13.1%]). In the NIS-Child public-use files, other races included Asian, American Indian or Alaska Native, Native Hawaiian or Pacific Islander, and other races as reported by the parent or caregiver.^16^

**Figure 1.**
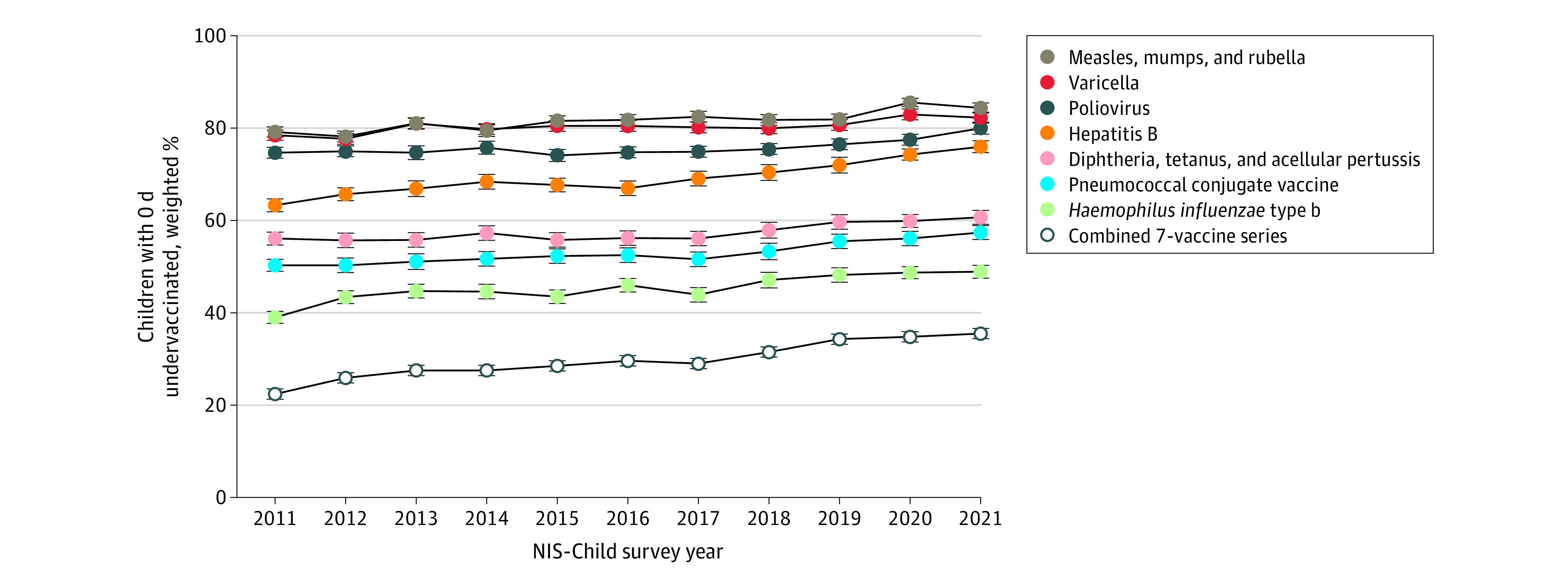
Prevalence of On-Time Receipt for 7 Vaccinations and for the Combined 7-Vaccine Series Among US Children Aged 0 to 19 Months, National Immunization Survey (NIS)–Child 2011-2021 On-time receipt is defined as 0 days undervaccinated. Significant changes in the prevalence of on-time vaccine receipt were observed for each vaccine series and for the combined 7-vaccine series (*P* < .001 for all). Statistical significance was assessed using log-linked binomial regression models with survey year as the independent variable to calculate the unadjusted mean annual change in the prevalence of on-time vaccine.

Completion of the combined 7-vaccine series by age 19 months increased from 52.2% (95% CI, 50.8%-53.5%) in the 2011 survey to 59.4% (95% CI, 57.9%-60.9%) in the 2021 survey (eTable 2 in Supplement 1). Approximately 1.3% of US children (3102 children) did not have any vaccinations from the combined 7-vaccine series by age 19 months; these children had an ADU of 501.3 days. Among the entire cohort, the median (IQR) ADU by age 19 months for the combined 7-vaccine series decreased from 22.3 (0.4-71.5) days in 2011 to 11.9 (0.0-55.5) days in 2021 (eTable 3 in Supplement 1). The percentage of children who received all vaccinations in the combined 7-vaccine series on time (ie, 0 days undervaccinated) increased from 22.5% (95% CI, 21.4%-23.6%) in 2011 to 35.6% (95% CI, 34.2%-37.0%) in 2021 (*P* < .001) (Figure 1). For specific vaccinations, the largest increases in the percentage of children who received all doses on time were for HepB and Hib vaccinations. The prevalence of on-time HepB vaccination increased from 63.4% (95% CI, 62.1%-64.8%) in 2011 to 76.1% (95% CI, 74.8%-77.4%) in 2021. On-time receipt of Hib vaccinations increased from 39.1% (95% CI, 37.8%-40.4%) to 49.0% (95% CI, 47.5%-50.4%) (eTable 4 in Supplement 1).

Multivariable models were conducted as complete case analyses and included 166 316 children (92.8% of starting cohort) for whom all covariate data were available. In a multivariable model without interaction terms, for every increase in survey year, there was a 3.4% mean increase in the prevalence of on-time vaccination for the combined 7-vaccine series (aPR for survey year, 1.034; 95% CI, 1.029-1.038) (eTable 5 in Supplement 1). Similar results were seen in a multivariable model limited to children who had completed the combined 7-vaccine series by age 19 months (eTable 6 in Supplement 1).

**Figure 2.**
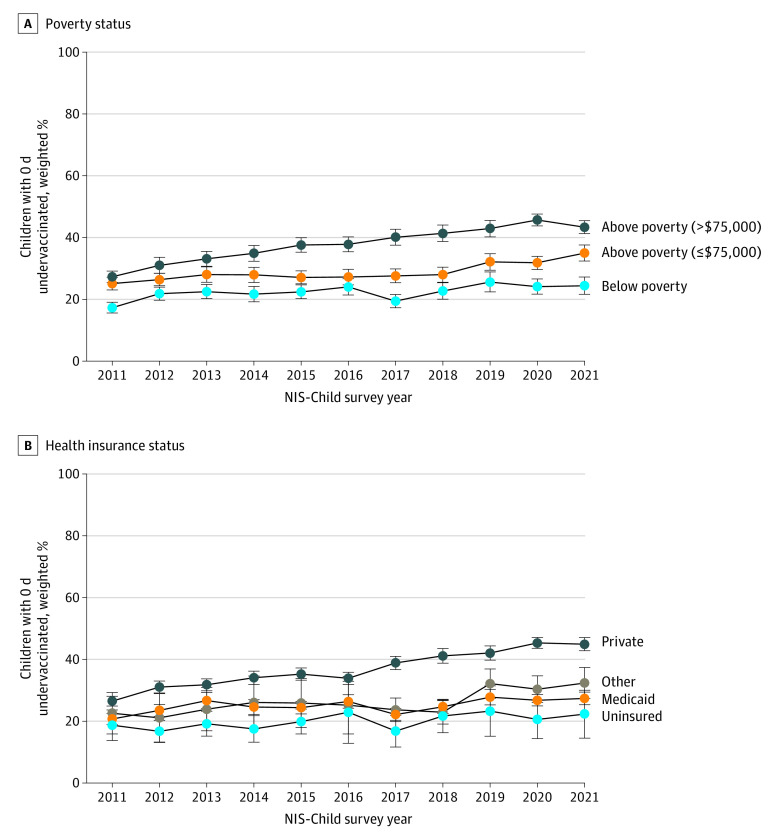
Prevalence of On-Time Receipt for the Combined 7-Vaccine Series, by Poverty and Health Insurance Status On-time receipt is defined as 0 days undervaccinated. A, Prevalence by poverty status based on US Census thresholds. Of the 179 154 US children with clinician-verified vaccination records in the 2011 to 2021 National Immunization Survey (NIS)–Child surveys, 6150 children were missing data on poverty status (6.0%, weighted). B, Prevalence by health insurance status. Among the total study sample, 4107 children were missing data on health insurance status (2.9%, weighted). If the child was enrolled in any form of Medicaid, alone or in combination with other types of health insurance coverage, the child was classified as any Medicaid in the NIS-Child survey. Other insurance includes the Children’s Health Insurance Program, Indian Health Service, Military, Tricare, Civilian Health and Medical Program of the Uniformed Services, or Civilian Health and Medical Program of the Department of Veterans Affairs (alone or in combination with private insurance).

Plots of the proportions of children who received the combined 7-vaccine series on time showed different rates of change by poverty status (Figure 2A) and insurance status (Figure 2B), with higher-income and privately insured children having higher rates of improvement in on-time vaccination than lower-income and non–privately insured children, respectively (eTable 7 in Supplement 1). In a multivariable model, there was a significant interaction between survey year and child’s poverty status. Compared with children living above the federal poverty level with more than $75 000 annual family income, children living above the poverty level with $75 000 or less annual family income had less pronounced increases in timeliness (aPR for interaction term, 0.983; 95% CI, 0.974-0.993; *P* < .001), as did children living below the federal poverty level (aPR for interaction term, 0.975; 95% CI, 0.964-0.987; *P* < .001) (Table 2) (full model results are shown in eTable 8 in Supplement 1). Overall, although children living above poverty with more than $75 000 annual family income had a 4.6% (95% CI, 4.0%-5.2%) mean annual increase in on-time vaccination, lower-income children had less marked mean annual increases (above poverty, ≤$75 000 annually, 2.8%; 95% CI, 2.0%-3.6%; below poverty, 2.0%; 95% CI, 1.0%-3.0%) (Table 3).

**Table 2.** Associations of Survey Year With Poverty Status (Model 1) and Health Insurance Status (Model 2) With On-Time Receipt of the Combined 7-Vaccine Series, National Immunization Survey–Child 2011-2021

Model and sociodemographic factors	Total study sample, No. (weighted %) (N = 179 154)	Outcome on-time vaccination (n = 59 445 [29.7%])^a^	
		Unadjusted PR (95% CI)^b^	Adjusted PR (95% CI)^c^
Model 1: interaction with poverty status			
Survey year	NA	1.041 (1.036-1.045)	1.046 (1.040-1.052)
Poverty status			
Above poverty, >$75 000 family income	74 479 (31.4)	[Reference]	NA
Above poverty, ≤$75 000 family income	58 961 (32.4)	0.742 (0.719-0.765)	NA
Below poverty	39 564 (30.2)	0.574 (0.552-0.596)	NA
Missing	6150 (6.0)	NA	NA
Survey year by poverty status			
Year, above poverty, >$75 000 family income	NA	NA	Reference
Year, above poverty, ≤$75 000 family income	NA	NA	0.983 (0.974-0.993)
Year, below poverty	NA	NA	0.975 (0.964-0.987)
Model 2: interaction with health insurance status			
Survey year	NA	1.041 (1.036-1.045)	1.046 (1.040-1.051)
Health insurance status			
Private insurance only	96 284 (43.1)	[Reference]	NA
Any Medicaid^d^	61 461 (44.0)	0.679 (0.659-0.700)	NA
Other insurance^e^	9073 (4.6)	0.733 (0.687-0.783)	NA
Uninsured	8229 (5.5)	0.522 (0.479-0.568)	NA
Missing	4107 (2.9)	NA	NA
Survey year by health insurance status			
Year, private insurance only	NA	NA	[Reference]
Year, any Medicaid^d^	NA	NA	0.971 (0.962-0.981)
Year, other insurance^e^	NA	NA	0.994 (0.970-1.018)
Year, uninsured	NA	NA	0.984 (0.955-1.014)

Abbreviations: NA, not applicable; PR, prevalence ratio.

^a^ On-time vaccination is defined as 0 days undervaccinated.

^b^ Unadjusted regression models presented in this table do not include an interaction term. These estimates represent the associations between year and on-time vaccination, poverty status and on-time vaccination, and health insurance status and on-time vaccination.

^c^ Adjusted for sex, race and ethnicity, US Census region, geographic mobility, number of clinicians administering vaccines, facility type, maternal education, number of children younger than 18 years in the household, and home ownership. Model 1 was also adjusted for health insurance status, and model 2 was also adjusted for poverty status. Owing to missing covariate data, 166 316 observations were included in multivariable analyses (90.3%, weighted), including 56 607 children with 0 days undervaccinated and complete covariate data. The following variables were missing data: facility type (2943 missing observations [1.1%, weighted]), poverty status (6150 missing observations [6.0%, weighted]), home ownership (524 missing observations [0.4%, weighted]), and insurance coverage (4107 missing observations [2.9%, weighted]).

^d^ If the child was enrolled in any form of Medicaid, alone or in combination with other types of health insurance coverage, the child was classified as any Medicaid in the National Immunization Survey–Child survey.

^e^ Other insurance includes the Children’s Health Insurance Program, Indian Health Service, Military, Tricare, Civilian Health and Medical Program of the Uniformed Services, or Civilian Health and Medical Program of the Department of Veterans Affairs (alone or in combination with private insurance).

**Table 3.** Mean Annual Change in On-Time Receipt of the Combined 7-Vaccine Series for Each Level of Poverty Status and Health Insurance Status

Variable	Unadjusted PR for survey year (95% CI)	Adjusted PR for survey year (95% CI)^a^	Adjusted mean annual change in on-time vaccine receipt, % (95% CI)^a^
Above poverty, >$75 000 family income	1.044 (1.038-1.050)	1.046 (1.040-1.052)	4.6 (4.0-5.2)
Above poverty, ≤$75 000 family income	1.027 (1.019-1.034)	1.028 (1.020-1.036)	2.8 (2.0-3.6)
Below poverty	1.022 (1.012-1.033)	1.020 (1.010-1.030)	2.0 (1.0-3.0)
Private insurance only	1.051 (1.045-1.056)	1.046 (1.040-1.051)	4.6 (4.0-5.1)
Any Medicaid^b^	1.018 (1.010-1.026)	1.016 (1.007-1.024)	1.6 (0.7-2.4)
Other insurance^c^	1.043 (1.018-1.068)	1.039 (1.015-1.064)	3.9 (1.5-6.4)
Uninsured	1.024 (0.996-1.054)	1.029 (0.999-1.060)	2.9 (0.0-6.0)

Abbreviation: PR, prevalence ratio.

^a^ The results shown were calculated in models that included an interaction term of survey year and either poverty status (top 3 rows) or insurance status (bottom 4 rows). Each row of results shows the estimates for survey year, modeled as a continuous variable, with that poverty status or insurance status level set as the reference group. Given that trends were linear on the basis of visual inspection, these results represent the unadjusted or adjusted mean annual change in on-time vaccination for that poverty status or health insurance status level. All multivariable models were adjusted for the following variables: sex, race and ethnicity, US Census region, geographic mobility, number of clinicians administering vaccines, facility type, maternal education, number of children younger than 18 years in the household, and home ownership. The poverty status models (top 3 rows) also adjusted for health insurance status; the health insurance status models (bottom 4 rows) also adjusted for poverty status. Missing covariate data were reported in Table 2.

^b^ If the child was enrolled in any form of Medicaid, alone or in combination with other types of health insurance coverage, the child was classified as any Medicaid in the National Immunization Survey–Child survey.

^c^ Other insurance includes the Children’s Health Insurance Program, Indian Health Service, Military, Tricare, Civilian Health and Medical Program of the Uniformed Services, or Civilian Health and Medical Program of the Department of Veterans Affairs (alone or in combination with private insurance).

In a separate multivariable model, there was a significant interaction between survey year and child’s health insurance status, with children with Medicaid insurance having significantly lower increases in on-time vaccination than privately insured children (aPR, 0.971; 95% CI, 0.962-0.981; *P* < .001) (Table 2) (full model results are shown in eTable 9 in Supplement 1). Although children with private health insurance had a 4.6% mean annual increase in on-time vaccination (95% CI, 4.0%-5.1%), children with Medicaid coverage had a 1.6% adjusted mean annual increase in on-time vaccination (95% CI, 0.7%-2.4%) (Table 3).

## Discussion

Our cross-sectional analyses of NIS-Child data found improvements in the percentage of children aged 0 to 19 months receiving all vaccinations in the combined 7-vaccine series on time, by ACIP-recommended ages, from the 2011 to 2021 surveys. Although there were improvements in on-time vaccination across all socioeconomic subgroups examined, the rate of improvement was greater for children from higher-income families and with private health insurance, compared with children from lower-income families or those with Medicaid insurance, respectively. As a result, disparities in vaccination timeliness by socioeconomic indicators widened over the 11-year period.

To date, monitoring progress toward childhood immunization goals has largely focused on measuring vaccination coverage, or completion of all recommended vaccine doses by a certain age, such as by age 24 months.^1,29^ In contrast to vaccination coverage, assessment of vaccination timeliness provides a more direct measure of adherence to national recommendations for the ages when children should receive vaccinations. Although prior studies have analyzed vaccination timeliness using 1 or 2 years of NIS-Child data, varying approaches to calculating days undervaccinated have made assessing changes over time challenging.^20,23,25,30^ By critically examining approaches used in prior studies, changes in ACIP recommendations, and CDSi documentation, we have established a method for calculating days undervaccinated that can be applied for future research and surveillance efforts, across data sources.

Although the Vaccines for Children program has been instrumental in removing financial cost as a barrier to vaccination, other challenges to accessing routine and timely preventive care remain for low-income and uninsured families.^31,32,33^ Most health system–level and clinic-level initiatives to improve access to immunization services have focused on proximate facilitators, such as contacting parents to let them know their child is coming due for vaccinations, making appointments easy to schedule, and offering walk-in immunization services.^34,35^ Our findings of widening socioeconomic disparities in timely vaccination suggest that such strategies may not have equitable reach across communities and that novel strategies may be needed to facilitate timely immunization services for lower-income children.^32^ Furthermore, to eliminate socioeconomic disparities in vaccination outcomes, policy approaches to address upstream causes of poverty and ensure equitable health insurance coverage for all children are needed.^36,37,38^

Tracking of vaccination timeliness throughout infancy can prompt earlier interventions to bring children up-to-date on vaccines, relative to waiting to measure vaccination coverage at age 2 years. Moving forward, measurement of timeliness should be incorporated into routine surveillance of vaccination uptake. Lack of timely vaccination is especially concerning when concentrated in communities and small geographic areas, because spatial clustering of undervaccination leads to greater risk of vaccine-preventable disease outbreaks. Although this current study illuminated national trends, surveillance at state and local levels is needed to identify specific subpopulations or geographic areas where interventions to improve on-time vaccination are needed. Although NIS-Child data can be used for some state-level surveillance, capabilities at smaller geographic levels are limited because of small sample sizes. However, all states maintain centralized immunization information systems, which consolidate vaccination records from across clinicians. These systems are increasingly being used for population-level surveillance of vaccination coverage.^39^ Future efforts should seek to advance the capabilities of immunization information systems for monitoring of vaccination timeliness within smaller geographic areas than what can be examined with NIS-Child data, and for informing public health responses to improve timely and routine childhood vaccination.^40^

### Limitations

This study has limitations that should be acknowledged. We were not able to assess the impacts of the COVID-19 pandemic on vaccination timeliness because birth year and vaccination dates are not available in the NIS-Child public-use files. Although some children surveyed at ages 19 to 35 months in the 2021 NIS-Child were due for vaccines after the start of the COVID-19 pandemic in the spring of 2020, most children in that survey year would have been due for vaccines before the pandemic’s start. Another limitation was that NIS-Child methods for collecting health insurance coverage information changed during the study period. Although the changes in survey questions likely did not impact categorization of private insurance, it may have impacted categorization of other insurance or Medicaid insurance. Furthermore, the NIS-Child does not collect data on why children fall behind or do not receive recommended vaccines. Our findings regarding disparities by socioeconomic status are consistent with other findings of similar disparities in child preventive health utilization, including disparities in rates of well-child visits.^33^ However, some of the lack of vaccination timeliness may have been due to other reasons unrelated to health care utilization and access, such as parental choice to delay vaccination.

## Conclusions

In this cross-sectional study of NIS-Child data collected from 2011 to 2021, we observed improvements in on-time vaccination among children aged 0 to 19 months in the US, overall and across poverty levels and health insurance types. However, rates of improvement differed by socioeconomic indicators. Disparities in timely vaccination signal the need for an increased focus on access to and quality of immunization services for lower-income families and children without private health insurance.

## Notes

Supplement 1. **eFigure.** Example of Calculating Days Undervaccinated **eTable 1.** Comparisons of Parameters for Measuring Days Undervaccinated for Early Childhood Vaccinations Across Information Sources **eAppendix.** Methods for Determining Parameters for Measuring Days Undervaccinated **eTable 2.** Vaccination Coverage for the Combined 7-Vaccine Series by Age 19 Months, National Immunization Survey-Child, 2011-2021 **eTable 3.** Distribution of Average Days Undervaccinated for the Combined 7-Vaccine Series and Days Undervaccinated for Nine Recommended Vaccinations, Among US Children by Age 19 Months, National Immunization Survey-Child 2011-2021 **eTable 4.** Prevalence of Children Who Received All Vaccine Doses On-Time (i.e., Zero Days Undervaccinated), for the Combined 7-Vaccine Series and for Nine Recommended Vaccinations, Among US Children by Age 19 Months, National Immunization Survey-Child 2011-2021 **eTable 5.** Associations Between Socioeconomic, Demographic, Household and Other Factors and On-Time Receipt of the Combined 7-Vaccine Series, National Immunization Survey-Child 2011-2021 **eTable 6.** Associations Between Socioeconomic, Demographic, Household and Other Factors and On-Time Receipt of the Combined 7-Vaccine Series, Among a Subcohort of US Children Who Completed the Combined 7-Vaccine Series by Age 19 Months, National Immunization Survey-Child 2011-2021 **eTable 7.** Prevalence of Children Who Received All Vaccine Doses On-Time (i.e., Zero Days Undervaccinated), for the Combined 7-Vaccine Series by Age 19 Months, by Poverty Status and Health Insurance Status, National Immunization Survey-Child 2011 and 2021 **eTable 8.** Associations Between Socioeconomic, Demographic, Household and Other Factors and On-Time Receipt of the Combined 7-Vaccine Series, Including Effect Modification by Poverty Status, National Immunization Survey-Child 2011-2021 **eTable 9.** Associations Between Socioeconomic, Demographic, Household and Other Factors and On-Time Receipt of the Combined 7-Vaccine Series, Including Effect Modification by Health Insurance Status, National Immunization Survey-Child 2011-2021
eReferences

Supplement 2.
Data Sharing Statement
