# Supplementary material for: Vaccination Timeliness Among US Children Aged 0-19 Months, National Immunization Survey–Child 2011-2021
Source: JAMA Netw Open. 2024 Apr 12;7(4):e246440. doi: 10.1001/jamanetworkopen.2024.6440 (PMC11015353; doi:10.1001/jamanetworkopen.2024.6440)
Supplement: Supplement 1. — eFigure. Example of Calculating Days Undervaccinated eTable 1. Comparisons of Parameters for Measuring Days Undervaccinated for Early Childhood Vaccinations Across Information Sources eAppendix. Methods for Determining Parameters for Measuring Days Undervaccinated eTable 2. Vaccination Coverage for the Combined 7-Vaccine Series by Age 19 Months, National Immunization Survey-Child, 2011-2021 eTable 3. Distribution of Average Days Undervaccinated for the Combined 7-Vaccine Series and Days Undervaccinated for Nine Recommended Vaccinations, Among US Children by Age 19 Months, National Immunization Survey-Child 2011-2021 eTable 4. Prevalence of Children Who Received All Vaccine Doses On-Time (i.e., Zero Days Undervaccinated), for the Combined 7-Vaccine Series and for Nine Recommended Vaccinations, Among US Children by Age 19 Months, National Immunization Survey-Child 2011-2021 eTable 5. Associations Between Socioeconomic, Demographic, Household and Other Factors and On-Time Receipt of the Combined 7-Vaccine Series, National Immunization Survey-Child 2011-2021 eTable 6. Associations Between Socioeconomic, Demographic, Household and Other Factors and On-Time Receipt of the Combined 7-Vaccine Series, Among a Subcohort of US Children Who Completed the Combined 7-Vaccine Series by Age 19 Months, National Immunization Survey-Child 2011-2021 eTable 7. Prevalence of Children Who Received All Vaccine Doses On-Time (i.e., Zero Days Undervaccinated), for the Combined 7-Vaccine Series by Age 19 Months, by Poverty Status and Health Insurance Status, National Immunization Survey-Child 2011 and 2021 eTable 8. Associations Between Socioeconomic, Demographic, Household and Other Factors and On-Time Receipt of the Combined 7-Vaccine Series, Including Effect Modification by Poverty Status, National Immunization Survey-Child 2011-2021 eTable 9. Associations Between Socioeconomic, Demographic, Household and Other Factors and On-Time Receipt of the Combined 7-Vaccine Series, Including Effect [file jamanetwopen-e246440-s001.pdf]

Supplemental Online Content

Newcomer SR, Michels SY, Albers AN, et al. Vaccination timeliness among US children aged 0-19 months, National Immunization Survey–Child 2011-2021. *JAMA Netw Open*. 2024;7(4):e246440. doi:10.1001/jamanetworkopen.2024.6440

eFigure. Example of Calculating Days Undervaccinated

eTable 1. Comparisons of Parameters for Measuring Days Undervaccinated for Early Childhood Vaccinations Across Information Sources

eAppendix. Methods for Determining Parameters for Measuring Days Undervaccinated

eTable 2. Vaccination Coverage for the Combined 7-Vaccine Series by Age 19 Months, National Immunization Survey-Child, 2011-2021

eTable 3. Distribution of Average Days Undervaccinated for the Combined 7-Vaccine Series and Days Undervaccinated for Nine Recommended Vaccinations, Among US Children by Age 19 Months, National Immunization Survey-Child 2011-2021

eTable 4. Prevalence of Children Who Received All Vaccine Doses On-Time (i.e., Zero Days Undervaccinated), for the Combined 7-Vaccine Series and for Nine Recommended Vaccinations, Among US Children by Age 19 Months, National Immunization Survey-Child 2011-2021

eTable 5. Associations Between Socioeconomic, Demographic, Household and Other Factors and On-Time Receipt of the Combined 7-Vaccine Series, National Immunization Survey-Child 2011-2021

eTable 6. Associations Between Socioeconomic, Demographic, Household and Other Factors and On-Time Receipt of the Combined 7-Vaccine Series, Among a Subcohort of US Children Who Completed the Combined 7-Vaccine Series by Age 19 Months, National Immunization Survey-Child 2011-2021

eTable 7. Prevalence of Children Who Received All Vaccine Doses On-Time (i.e., Zero Days Undervaccinated), for the Combined 7-Vaccine Series by Age 19 Months, by Poverty Status and Health Insurance Status, National Immunization Survey-Child 2011 and 2021

eTable 8. Associations Between Socioeconomic, Demographic, Household and Other Factors and On-Time Receipt of the Combined 7-Vaccine Series, Including Effect Modification by Poverty Status, National Immunization Survey-Child 2011-2021

eTable 9. Associations Between Socioeconomic, Demographic, Household and Other Factors and On-Time Receipt of the Combined 7-Vaccine Series, Including Effect Modification by Health Insurance Status, National Immunization Survey-Child 2011-2021

eReferences

This supplemental material has been provided by the authors to give readers additional information about their work.

© 2024 Newcomer SR et al. *JAMA Network Open.*

eFigure. Example of Calculating Days Undervaccinated

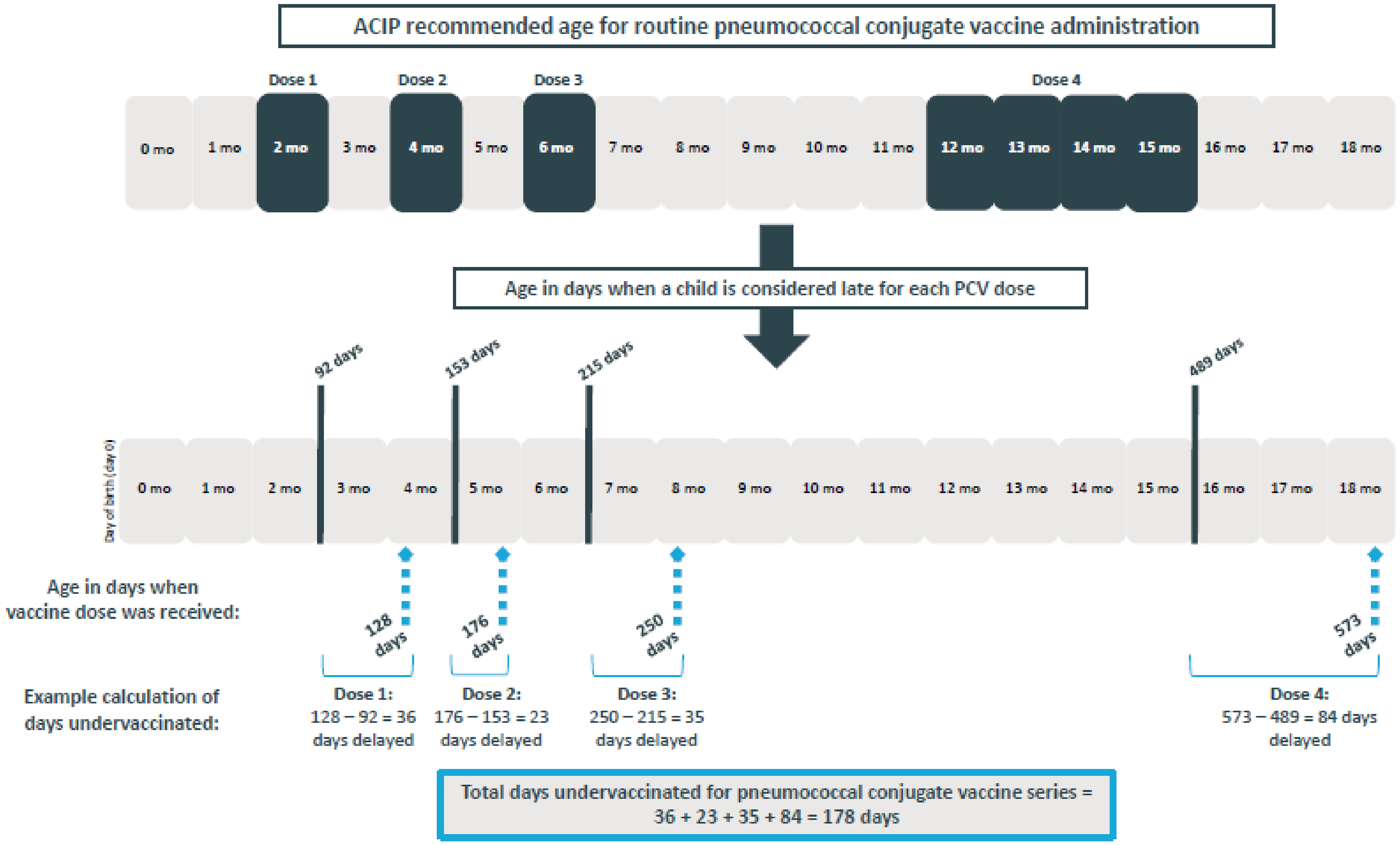

© 2024 Newcomer SR et al. *JAMA Network Open.*

eTable 1. Comparisons of Parameters for Measuring Days Undervaccinated for Early Childhood Vaccinations Across Information Sources

Parameters for recommended age for routine administration and minimum acceptable age of receipt

	Recommended age for routine administration					Minimum acceptable age of receipt[a]				
	ACIP	CDSi	Previous studies[b]			ACIP	CDSi	Previous studies[b]		
**Vaccination dose**			Luman et al., 2005	Opel et al., 2011 & Kurosky et al., 2016	Glanz et al., 2013; Newcomer et al. 2021; & Freeman et al., 2022			Luman et al., 2005	Opel et al., 2011 & Kurosky et al., 2016	Glanz et al., 2013; Newcomer et al. 2021; & Freeman et al., 2022
**HepB**										
Dose 1	0 mo	0 d	0-2 mo	Birth (0-3 d)	0-2 mo	Birth	0 d	Birth	Birth	0 d
Dose 2	1-2 mo	1 mo	1-4 mo	1-2 mo	1-4 mo	4 wk	4 wk	4 wk	28 d (4 wk)	24 d
Dose 3	6-18 mo	6 mo	6-18 mo	6-18 mo	6-18 mo	24 wk	24 wk	6 mo	168 d (24 wk)	176/164 d[c]
**DTaP**										
Dose 1	2 mo	2 mo	2 mo	2 mo	2 mo	6 wk	6 wk	6 wk	42 d (6 wk)	38 d
Dose 2	4 mo	4 mo	4 mo	4 mo	4 mo	--	10 wk	10 wk	70 d (10 wk)	66 d
Dose 3	6 mo	6 mo	6 mo	6 mo	6 mo	--	14 wk	14 wk	98 d (14 wk)	94 d
Dose 4	15-18 mo	15 mo	15-18 mo	15-18 mo	15-18 mo	12 mo	12 mo	12 mo	365 d (12 mo)	361 d
**Hib**										
Dose 1	2 mo	2 mo	2 mo	2 mo	2 mo	6 wk	6 wk	6 wk	42 d (6 wk)	38 d
Dose 2	4 mo	4 mo	4 mo	4 mo	4 mo	--	10 wk	10 wk	70 d (10 wk)	66 d
Dose 3	6 mo	6 mo	6 mo	6 mo	6 mo	--	14 wk	14 wk	98 d (14 wk)	94 d
Dose 4	12-15 mo	12 mo	12-15 mo	12-15 mo	12-15 mo	--	12 mo	12 mo	12 mo	361 d
**PCV**										
Dose 1	2 mo	2 mo	--	2 mo	2 mo	6 wk	6 wk	--	6 wk	38 d
Dose 2	4 mo	4 mo	--	4 mo	4 mo	--	10 wk	--	10 wk	66 d
Dose 3	6 mo	6 mo	--	6 mo	6 mo	--	14 wk	--	14 wk	94 d
Dose 4	12-15 mo	12 mo	--	12-15 mo	12-15 mo	--	12 mo	--	12 mo	361 d
**IPV**										
Dose 1	2 mo	2 mo	2 mo	2 mo	2 mo	6 wk	6 wk	6 wk	42 d (6 wk)	38 d
Dose 2	4 mo	4 mo	4 mo	4 mo	4 mo	--	10 wk	10 wk	70 d (10 wk)	66 d
Dose 3	6-18 mo	6 mo	6-18 mo	6-18 mo	6-18 mo	--	14 wk	14 wk	98 d (14 wk)	94 d
**MMR**										
Dose 1	12-15 mo	12 mo	12-15 mo	12-15 mo	12-15 mo	12 mo	12 mo	12 mo	365 d (12 mo)	361 d
**VAR**										
Dose 1	12-15 mo	12 mo	12-18 mo	12-15 mo	12-15 mo	12 mo	12 mo	12 mo	365 d (12 mo)	361 d
**RV**										
Dose 1	2 mo	2 mo	--	2 mo	2 mo	6 wk	6 wk	--	6 wk	38 d
Dose 2	4 mo	4 mo	--	4 mo	4 mo	--	10 wk	--	10 wk	66 d
Dose 3	6 mo	6 mo	--	6 mo	6 mo	--	14 wk	--	14 wk	94 d
**HepA**										
Dose 1	12 mo	12 mo	--	--	--	12 mo	12 mo	--	--	--
Dose 2	--	18 mo	--	--	--	--	18 mo	--	--	--

© 2024 Newcomer SR et al. *JAMA Network Open.*

Parameters for minimum acceptable interval between doses and days undervaccinated count initiation

Vaccination dose	Minimum acceptable interval between doses[a]					Days undervaccinated count initiation		
	ACIP	CDSi	Previous studies[b]			Previous studies[b]		
			Luman et al., 2005	Opel et al., 2011 & Kurosky et al., 2016	Glanz et al., 2013; Newcomer et al. 2021; & Freeman et al., 2022	Luman et al., 2005	Opel et al., 2011 & Kurosky et al., 2016	Glanz et al., 2013; Newcomer et al. 2021; & Freeman et al., 2022
**HepB**								
Dose 1	--		--	--	--	93 d	32 d/4 d[d]	93 d
Dose 2	4 wk	4 wk	4 wk	28 d (4 wk)	24 d	154 d	93 d	154 d
Dose 3[e]	8 wk	8 wk	8 wk	56 d (8 wk)	38 d	580 d	580 d	580 d
Dose 1–3	16 wk	16 wk	--	--	--	--	--	--
**DTaP**								
Dose 1	--	--	--	--	--	93 d	93 d	93 d
Dose 2	4 wk	4 wk	4 wk	28 d (4 wk)	24 d	154 d	154 d	154 d
Dose 3	4 wk	4 wk	4 wk	28 d (4 wk)	24 d	215 d	215 d	215 d
Dose 4	4mo/6 mo[f]	4 mo	6 mo	180 d (6 mo)	179 d	580 d	580 d	580 d
**Hib**								
Dose 1	--	--	--	--	--	93 d	93 d	93 d
Dose 2	4 wk	4 wk	4 wk	28 d (4 wk)	24 d	154 d	154 d	154 d
Dose 3	4 wk	4 wk	4 wk	28 d (4 wk)	24 d	215 d	215 d	215 d
Dose 4	8 wk	8 wk	8 wk	6 mo	52 d	489 d	489 d	580 d
**PCV**								
Dose 1	--	--	--	--	--	--	93 d	93 d
Dose 2	4 wk	4 wk	--	8 wks	24 d	--	154 d	154 d
Dose 3	4 wk	4 wk	--	8 wks	24 d	--	215 d	215 d
Dose 4	8 wk	8 wk	--	6 mo	52 d	--	489 d	580 d
**IPV**								
Dose 1	--	--	--	--	--	93 d	93 d	93 d
Dose 2	4 wk	4 wk	4 wk	28 d (4 wk)	24 d	154 d	154 d	154 d
Dose 3	4 wk	4 wk	4 wk	28 d (4 wk)	24 d	580 d	580 d	580 d
**MMR**								
Dose 1	--	--	--	--	--	489 d	489 d	489 d
**VAR**								
Dose 1	--	--	--	--	--	580 d	489 d	489 d
**RV**[g]								
Dose 1	--	--	--	--	--	--	93 d	93 d
Dose 2	4 wk	4 wk	--	2 mo	24 d	--	154 d	154 d
Dose 3	4 wk	4 wk	--	2 mo	24 d	--	215 d	215 d

© 2024 Newcomer SR et al. *JAMA Network Open.*

**HepA**								
Dose 1	--	--	--	--	--	--	--	--
Dose 2	6 mo	6 mo	--	--	--	--	--	--

--, Not included in source documentation; ACIP, Advisory Committee on Immunization Practices; CDSi, Clinical Decision Support for Immunization; d, days; DTaP, Diphtheria, tetanus, acellular pertussis; Hib, *Haemophilus influenzae* type b; HepA, Hepatitis A; HepB, Hepatitis B; IPV, inactivated poliovirus vaccine; MMR, measles, mumps, rubella; mo, months; PCV, pneumococcal conjugate vaccine; RV, rotavirus; VAR, varicella; wk, week

[a] Allow for 4-day grace period. Opel et al.[1] stated in their methods that the 4-day grace period was taken into account in their programming and final calculations.
[b] Previous studies include: Luman et al., 2005 (vaccines studied: HepB, DTaP, Hib, IPV, MMR, VAR)[2]; Opel et al., 2011 (vaccines studied: HepB, DTaP, Hib (3-dose series only), IPV, MMR, VAR)[1]; Glanz et al., 2013 (vaccines studied: HepB, DTaP, Hib, PCV, IPV, MMR, VAR, RV)[3]; Kurosky et al., 2016 (vaccines studied: HepB, DTaP, Hib, PCV, IPV, MMR, VAR, RV)[4]; Newcomer et al., 2021 (vaccines studied: HepB, DTaP, Hib, PCV, IPV, MMR, VAR)[5]; Freeman et al., 2022 (vaccines studied: HepB, DTaP, Hib, PCV, IPV, MMR, VAR).[6]
[c] Glanz et al. considered 176 days (6 months x 30 days = 180 days – 4-day grace period = 176 days) the minimum acceptable age of HepB vaccination dose 3. Freeman et al. and Newcomer et al. changed the minimum age to 164 days to account for ACIP guidelines that specify HepB dose 3 should be administered at age 24 weeks (24 weeks x 7 days = 168 days – 4-day grace period = 164 days).
[d] Opel et al. set the days undervaccinated count initiation parameter at age 32 days for the first dose of hepatitis B vaccination[1]; Kurosky et al. set the parameter at age 4 days.[4]
[e] When 4 doses are administered, substitute "dose 4" for "dose 3" in these calculations.
[f] In 2018, the ACIP published additional guidance regarding the minimum acceptable interval between the 3rd and 4th DTaP doses, which clarified that 6 months is the minimum recommended interval for prospective administration; however, a four-month interval is acceptable in retrospective evaluation.
[g] Per ACIP recommendations, the maximum age for RV dose 1 is 14 weeks, 6 days. Children are not recommended to start the series on or after age 15 weeks, 0 days. The maximum age for the final dose is 8 months, 0 days.

© 2024 Newcomer SR et al. *JAMA Network Open.*

eAppendix. Methods for Determining Parameters for Measuring Days Undervaccinated

Determining age in days for parameters

To date, the main data sources used to measure days undervaccinated have been National Immunization Survey-Child (NIS-Child), electronic health record (EHR), and state and jurisdictional immunization information system (IIS) data.[7] While EHR and IIS records may include birth and vaccination dates, these variables may be deidentified for research purposes by calculating age in days for each vaccination and then removing dates. Public use NIS-Child data files only contain children's age in days for each vaccine received; dates are not available. Therefore, we sought to determine parameters for measuring days undervaccinated using age-in-days variables.

In both Advisory Committee for Immunization Practices (ACIP) and Clinical Decision Support for Immunizations (CDSi) documentation, the minimum acceptable age of vaccine receipt is provided in weeks in age, with the exception of measles-mumps-rubella (MMR), varicella, and Hepatitis A (HepA) vaccines, for which 12 months is provided as the minimum age. Similarly, in both ACIP and CDSi documentation, the recommended interval between doses of multi-dose vaccinations is provided in weeks, with the exception the recommended interval between the 3rd and 4th doses of DTaP, which is provided in months. To calculate the minimum age of receipt and minimum acceptable interval between doses in days, ages or intervals provided in weeks were multiplied by 7 days. As allowable per ACIP recommendations, a 4-day grace period was subtracted.[8] Similarly, to calculate the minimum age in days for MMR, varicella, and HepA vaccines, and the minimum interval between the 3rd and 4th doses of DTaP vaccines, we determined the minimum number of days necessary to achieve the minimum age or interval (which was provided in months), and subtracted 4 days.

The ACIP schedule provides a one-month or multiple-month recommended interval for when each vaccine dose should be given. Once that interval has elapsed, a child is considered late in receiving the vaccine. To determine the age in days when a child should first be considered late for a vaccine and begin accumulating days undervaccinated (i.e., the days undervaccinated count initiation parameter), previous studies have multiplied the number of months by either 30, 30.5, or 31 days.[1-6] However, when the month and year a child was born is not available in the source data, there may be variability in this calculation as there is a range of possible ages in days. For example, the first dose of PCV is recommended at age 2 months. The day of birth is considered day 0. Since this first PCV dose is considered late the day the child turns 3 months old, the child could be as young as age 89 days if born in February or as old as age 92 days if born in July. To be consistent across milestone ages and avoid misclassifying children as undervaccinated, we used the oldest possible age in days a child could be to initiate the days undervaccinated count. To achieve this consistency, we assumed the child was born on July 1st and the first year of life included a leap year, such that the following February (when the child turns age 7 months) has 29 days. The months of July and August both have 31 days. Similarly, December and January each have 31 days, so this calculation yields the oldest possible age in days a child could be and have received recommended vaccine doses on-time.

Updates to ACIP documentation

In review of the 2011-2022 ACIP schedules and accompanying documentation, we identified two changes that affected measurement of days undervaccinated.

Starting with the 2012 schedule, guidance was added specifying that the minimum acceptable interval between the 1st and 3rd HepB doses is 16 weeks, which we interpreted as 108 days (16 weeks x 7 days=112 days–4-day grace period=108 days). Of the nine routine childhood vaccine series we examined in this project, hepatitis B (HepB) is the only one where the ACIP schedule specified minimal intervals between non-subsequent doses. Since this documentation was added as clarification early in the study period, we applied the minimum interval parameter of 108 days between the 1st and 3rd HepB doses across all years of study data (2011-2021).

Starting with the 2018 schedule, additional guidance was added regarding the minimum acceptable interval between the 3rd and 4th DTaP vaccination doses. This guidance clarified that six months is the minimum recommended interval for prospective administration; however, a four-month interval is acceptable in retrospective evaluation. In other words, if it is determined that an already-administered 4th DTaP dose was at least four months after the prior dose, it does not need to be re-administered. Since this clarification allowed for a less strict minimum interval between the 3rd and 4th DTaP vaccine does, we applied it across all years of study data (2011-2021).

Differences in parameters for measuring days undervaccinated across information sources

In eTable1, we provide side-by-side comparisons of parameters identified from three information sources: ACIP schedules and accompanying documentation, CDSi documentation, and previous studies. ACIP and CDSi parameters were similar; however, there were differences in days undervaccinated parameters used for some vaccines in prior studies.

Across information sources, there were multiple differences for the parameters used to calculate days undervaccinated for the Hepatitis B vaccine. The ACIP recommends that medically stable children weighing ≥2,000 grams receive a monovalent HepB vaccination within 24 hours of birth, and all other children should receive the vaccination by age 1 months or at hospital discharge, whichever is first.[8] Prior studies have initiated the days undervaccinated count for the first HepB vaccine dose at age 4 days[4], 32 days[1], or 93 days.[3,5,6] For this study, we set the days undervaccinated count initiation parameter for the dose 1 of the Hepatitis B vaccine to age 31 days, since this is the oldest a child can be in age in days when turning 1 month old, assuming that birth is day 0.

ACIP and CDSi documentation both state that the minimum age of receipt of the 3rd HepB vaccine dose is 24 weeks; however, Luman et al. expressed this minimum acceptable age as 6 months. As a result, the minimum acceptable age in days for the 3rd HepB

© 2024 Newcomer SR et al. *JAMA Network Open.*

vaccine dose differed across studies, likely due to methodological differences in converting months to days versus weeks to days. For this study, and similar to other studies[5,6], we set the parameter of the minimum acceptable age of receipt of dose 3 of the Hepatitis B vaccine to 164 days (24 weeks x 7 days=168 days–4-day grace period=164 days).

Additionally, the minimum acceptable interval between HepB vaccine dose 2 and dose 3 differed across studies. The package insert for the combination DTaP-IPV-HepB vaccine (Pediarix®, GlaxoSmithKline) states that vaccine doses should be administered "at intervals of 6-8 weeks, preferably 8 weeks"[9] (38-52 days; 6 weeks x 7 days=42 days–4-day grace period=38 days and 8 weeks x 7 days=56 days–4-day grace period=52 days). Some prior studies calculated the minimum acceptable interval between HepB vaccine dose 2 and 3 to be 38 days to account for the use of the combination DTaP-IPV-HepB vaccines.[3,5,6] However, ACIP and CDSi documentation specifies an 8 week interval between doses 2 and 3, and other prior studies used 56 days, minus a 4-day grace period, for this interval.[1,4] Therefore, for this study, we established this minimum acceptable interval between doses 2 and 3 of the Hepatitis B vaccine to be 52 days between dose 2 and dose 3.

Across previous studies, there was also variation in the parameters applied for the *Haemophilus* influenzae (Hib) and pneumococcal conjugate (PCV) vaccinations. The final doses of both Hib and pneumococcal vaccine series are recommended by the ACIP at ages 12-15 months, and a child would be considered late on the day they turned age 16 months. Some prior studies set the days undervaccinated count to initiate at age 489 days (approximately age 16 months) for the final doses of both Hib and pneumococcal vaccinations[1,4]; however, other studies used age 580 days (approximately age 19 months)[3,5,6] to account for the DTaP-IPV-Hib combination vaccine (Pentacel®, Sanofi Pasteur), which the package insert state should be administered at 2,4,6, and 15-18 months.[10] Because pneumococcal, DTaP-containing, and Hib-containing vaccines are often concomitantly administered, these studies also initiated the pneumococcal vaccine series days undervaccinated count at age 19 months.[3,5,6] However, since the ACIP recommendations are for the fourth doses to be administered at 12-15 months, we initiated the days undervaccination calculation for the booster vaccination doses of both Hib and PCV at age 489 days for this analysis.

In 2007, the ACIP recommended age for varicella vaccination changed from 12-18 months to 12-15 months.[11] Luman and colleagues published their seminal work on vaccination timeliness in 2005[2], prior to this change. Subsequent studies updated the days undervaccinated count initiation parameter to account for the change in ACIP recommendations.[1,3-6] Similar to other previous studies, in this study we began days undervaccinated count calculations at age 489 days.

Parameters for measuring days undervaccinated for the first dose of hepatitis A vaccination

To our knowledge, parameters for calculating days undervaccinated for HepA vaccination have not been previously published. During the study period, the ACIP recommended a two dose Hepatitis A series for children ages 12-23 months (inclusive), with a minimum interval between doses of 6 months. Since we were measuring days undervaccinated for children ages 0-19 months, we only established parameters for the first dose of HepA vaccination. Per ACIP recommendations, the minimum acceptable age of receipt for dose 1 is age 12 months (365 days-4-day grace period= 361 days). Given that a minimum of 6 months is required between the two recommended HepA doses (and allowing for a 4-day grace period), to complete the 2-dose HepA series by the child's second birth day, children must receive the first dose before age 550 days. Therefore, the days undervaccinated count initiation parameter for the first dose of Hepatitis A vaccination was set to age 550 days in this study.

© 2024 Newcomer SR et al. *JAMA Network Open.*

eTable 2. Vaccination Coverage for the Combined 7-Vaccine Series by Age 19 Months, National Immunization Survey-Child, 2011-2021

	NIS-Child Survey Year										
	**2011 n=19,144**	**2012 n=16,687**	**2013 n=13,611**	**2014 n=14,893**	**2015 n=15,167**	**2016 n=14,988**	**2017 n=15,333**	**2018 n=15,657**	**2019 n=16,365**	**2020 n=19,342**	**2021 n=17,967**
**Vaccination(s)**	**Weighted %** (95% CI)										
**Combined 7-vaccine series**	**52.2%** (50.8%-53.5%)	**54.3%** (52.8%-55.8%)	**54.9%** (53.2%-56.5%)	**57.2%** (55.6%-58.8%)	**57.7%** (56.2%-59.2%)	**57.2%** (55.6%-58.8%)	**57.1%** (55.4%-58.7%)	**59.0%** (57.2%-60.7%)	**61.6%** (59.9%-63.3%)	**61.4%** (60.1%-62.8%)	**59.4%** (57.9%-60.9%)
**HepB** ≥3 doses	**85.8%** (84.9%-86.8%)	**85.4%** (84.4%-86.4%)	**86.4%** (85.2%-87.6%)	**87.9%** (86.8%-88.9%)	**87.8%** (86.7%-88.8%)	**86.4%** (85.1%-87.6%)	**87.8%** (86.6%-89.0%)	**89.0%** (87.9%-90.0%)	**88.8%** (87.6%-90.0)	**90.0%** (89.2%-90.8%)	**90.6%** (89.7%-91.5%)
**DTaP** ≥4 doses	**69.5%** (68.3%-70.8%)	**68.1%** (66.7%-69.5%)	**67.8%** (66.2%-69.3%)	**69.8%** (68.3%-71.3%)	**69.5%** (68.1%-71.0%)	**68.7%** (67.1%-70.2%)	**69.2%** (67.7%-70.8%)	**69.3%** (67.6%-71.0%)	**71.5%** (70.0%-73.1%)	**72.2%** (70.9%-73.4%)	**71.1%** (69.7%-72.5%)
**Hib** ≥3 or ≥4 doses[a]	**68.7%** (67.5%-70.0%)	**71.4%** (70.0%-72.7%)	**72.1%** (70.6%-73.6%)	**71.9%** (70.4%-73.4%)	**72.6%** (71.2%-74.0%)	**72.1%** (70.6%-73.6%)	**72.7%** (71.2%-74.1%)	**73.2%** (71.6%-74.8%)	**73.8%** (72.2%-75.4%)	**74.7%** (73.5%-75.9%)	**71.8%** (70.5%-73.2%)
**PCV** ≥4 doses	**75.7%** (74.6%-76.9%)	**74.4%** (73.1%-75.7%)	**75.5%** (74.0%-76.9%)	**75.8%** (74.3%-77.3%)	**77.6%** (76.3%-78.9%)	**75.6%** (74.1%-77.1%)	**76.9%** (75.5%-78.2%)	**76.1%** (74.4%-77.7%)	**77.3%** (75.8%-78.9%)	**78.6%** (77.5%-79.8%)	**78.7%** (77.4%-79.9%)
**IPV** ≥3 doses	**90.8%** (90.0%-91.5%)	**90.3%** (89.5%-91.2%)	**90.3%** (89.3%-91.4%)	**91.0%** (90.0%-92.0%)	**91.3%** (90.5%-92.1%)	**89.2%** (88.0%-90.3%)	**90.3%** (89.2%-91.4%)	**91.3%** (90.4%-92.1%)	**90.1%** (89.0%-91.3%)	**92.2%** (91.5%-93.0%)	**92.2%** (91.3%-93.0%)

© 2024 Newcomer SR et al. *JAMA Network Open.*

	NIS-Child Survey Year										
	**2011 n=19,144**	**2012 n=16,687**	**2013 n=13,611**	**2014 n=14,893**	**2015 n=15,167**	**2016 n=14,988**	**2017 n=15,333**	**2018 n=15,657**	**2019 n=16,365**	**2020 n=19,342**	**2021 n=17,967**
	**Weighted %** (95% CI)										
**MMR** ≥1 dose	**87.2%** (86.3%-88.1%)	**86.1%** (85.1%-87.2%)	**87.5%** (86.4%-88.6%)	**86.8%** (85.7%-88.0%)	**88.0%** (87.1%-89.0%)	**87.4%** (86.3%-88.5%)	**88.3%** (87.4%-89.3%)	**88.1%** (87.0%-89.2%)	**87.5%** (86.2%-88.7%)	**90.2%** (89.4%-91.0%)	**89.1%** (88.1%-90.0%)
**VAR** ≥1 dose	**86.0%** (85.1%-86.9%)	**85.7%** (84.6%-86.7%)	**86.9%** (85.8%-88.0%)	**86.1%** (85.0%-87.2%)	**86.7%** (85.7%-87.7%)	**86.4%** (85.2%-87.5%)	**87.0%** (85.9%-88.0%)	**87.2%** (86.0%-88.3%)	**86.3%** (85.1%-87.6%)	**88.8%** (87.9%-89.6%)	**87.7%** (86.7%-88.8%)

CI, confidence interval; DTaP, diphtheria-tetanus-acellular pertussis; HepB, hepatitis B; Hib, *Haemophilus* influenzae type b; IPV, inactivated poliovirus; MMR, measles-mumps-rubella; NIS-Child, National Immunization Survey-Child; PCV, pneumococcal conjugate vaccine; VAR, varicella

[a] Number of recommended vaccine doses depends on manufacturing brand type.

© 2024 Newcomer SR et al. *JAMA Network Open.*

eTable 3. Distribution of Average Days Undervaccinated for the Combined 7-Vaccine Series and Days Undervaccinated for Nine Recommended Vaccinations, Among US Children by Age 19 Months, National Immunization Survey-Child 2011-2021

Vaccination(s)	NIS-Child Survey Year										
	**2011 n=19,144**	**2012 n=16,687**	**2013 n=13,611**	**2014 n=14,893**	**2015 n=15,167**	**2016 n=14,988**	**2017 n=15,333**	**2018 n=15,657**	**2019 n=16,365**	**2020 n=19,342**	**2021 n=17,967**
	**Average days undervaccinated**										
**Combined 7-vaccine series** *Range: 0-501.3*											
Mean (SE)	68.4 (1.5)	70.9 (1.8)	67.4 (2.2)	65.9 (1.9)	64.9 (1.6)	69.6 (2.0)	68.2 (2.0)	66.3 (2.3)	66.1 (2.4)	58.7 (1.6)	57.3 (1.7)
Median (IQR)	*22.3 (0.4-71.5)*	*17.9 (0.0-73.2)*	*17.3 (0.0-66.2)*	*17.2 (0.0-69.2)*	*17.1 (0.0-69.3)*	*17.1 (0.0-69.2)*	*17.1 (0.0-69.2)*	*14.6 (0.0-63.7)*	*12.7 (0.0-59.4)*	*12.1 (0.0-56.5)*	*11.9 (0.0-55.5)*
	**Days undervaccinated**										
**HepB** *Range: 0-551*											
Mean (SE)	56.7 (1.7)	60.0 (2.1)	53.9 (2.3)	50.8 (2.0)	49.7 (1.8)	56.0 (2.2)	53.8 (2.3)	49.7 (2.4)	48.0 (2.5)	41.8 (1.6)	37.6 (1.7)
Median (IQR)	*0.0 (0.0-60.4)*	*0.0 (0.0-54.0)*	*0.0 (0.0-34.1)*	*0.0 (0.0-30.2)*	*0.0 (0.0-31.4)*	*0.0 (0.0-44.0)*	*0.0 (0.0-18.6)*	*0.0 (0.0-11.7)*	*0.0 (0.0-1.61)*	*0.0 (0.0-0.2)*	*0.0 (0.0-0.0)*
**DTaP** *Range: 0-490*											
Mean (SE)	54.4 (1.8)	60.1 (2.0)	60.4 (2.5)	57.4 (2.3)	56.6 (1.9)	62.3 (2.4)	61.0 (2.3)	59.6 (2.6)	60.3 (2.6)	53.7 (1.8)	50.6 (2.0)
Median (IQR)	*0.0 (0.0-14.4)*	*0.0 (0.0-19.2)*	*0.0 (0.0-19.5)*	*0.0 (0.0-15.3)*	*0.0 (0.0-17.5)*	*0.0 (0.0-19.8)*	*0.0 (0.0-20.5)*	*0.0 (0.0-15.2)*	*0.0 (0.0-12.3)*	*0.0 (0.0-9.9)*	0.0 *(0.0-3.3)*
**Hib** *Range: 0-490*											
Mean (SE)	101.2 (2.0)	92.8 (2.1)	88.0 (2.5)	89.7 (2.5)	90.8 (2.1)	92.3 (2.5)	92.6 (2.5)	90.9 (3.0)	89.9 (2.9)	82.6 (2.0)	87.8 (2.3)
Median (IQR)	*37.2 (0.0-101.5)*	*17.1 (0.0-92.9)*	*11.2 (0.0-92.7)*	*12.1 (0.0-92.8)*	*13.1 (0.0-92.9)*	*8.4 (0.0-92.8)*	*12.0 (0.0-92.9)*	*5.8 (0.0-92.8)*	*2.5 (0.0-92.6)*	*1.8 (0.0-92.6)*	*1.9 (0.0-92.7)*
**PCV** *Range: 0-490*											
Mean (SE)	88.1 (2.0)	95.0 (2.3)	91.1 (2.7)	88.7 (2.6)	84.6 (2.2)	92.5 (2.8)	89.6 (2.7)	87.6 (3.1)	85.3 (2.8)	79.1 (2.1)	73.6 (2.2)
Median (IQR)	*0.0 (0.0-92.8)*	*0.0 (0.0-101.3)*	*0.0 (0.0-92.8)*	*0.0 (0.0-92.9)*	*0.0 (0.0-92.6)*	*0.0 (0.0-92.8)*	*0.0 (0.0-92.8)*	*0.0 (0.0-92.8)*	*0.0 (0.0-92.5)*	*0.0 (0.0-92.2)*	*0.0 (0.0-92.1)*

© 2024 Newcomer SR et al. *JAMA Network Open.*

Vaccination(s)	NIS-Child Survey Year 2011 n=19,144	2012 n=16,687	2013 n=13,611	2014 n=14,893	2015 n=15,167	2016 n=14,988	2017 n=15,333	2018 n=15,657	2019 n=16,365	2020 n=19,342	2021 n=17,967
	Days undervaccinated										
**IPV** *Range: 0-490*											
Mean (SE)	36.4 (1.4)	40.6 (1.7)	40.4 (2.2)	37.1 (1.8)	38.9 (1.6)	42.1 (1.9)	41.4 (2.0)	40.5 (2.1)	43.4 (2.5)	35.0 (1.5)	33.2 (1.6)
Median (IQR)	*0.0 (0.0-0.1)*	*0.0 (0.0-0.0)*	*0.0 (0.0-0.1)*	*0.0 (0.0-0.0)*	*0.0 (0.0-0.5)*	*0.0 (0.0-0.0)*	*0.0 (0.0-0.0)*	*0.0 (0.0-0.0)*	*0.0 (0.0-0.0)*	*0.0 (0.0-0.0)*	*0.0 (0.0-0.0)*
**MMR** *Range: 0-93*											
Mean (SE)	15.2 (0.4)	16.1 (0.5)	14.1 (0.5)	15.1 (0.5)	13.6 (0.5)	14.0 (0.5)	13.4 (0.5)	13.6 (0.6)	13.8 (0.6)	10.8 (0.4)	12.0 (0.5)
Median (IQR)	*0.0 (0.0-0.0)*	*0.0 (0.0-0.0)*	*0.0 (0.0-0.0)*	*0.0 (0.0-0.0)*	*0.0 (0.0-0.0)*	*0.0 (0.0-0.0)*	*0.0 (0.0-0.0)*	*0.0 (0.0-0.0)*	*0.0 (0.0-0.0)*	*0.0 (0.0-0.0)*	*0.0 (0.0-0.0)*
**VAR** *Range: 0-93*											
Mean (SE)	16.2 (0.5)	16.8 (0.5)	14.6 (0.5)	15.7 (0.5)	15.0 (0.5)	15.1 (0.6)	15.1 (0.5)	15.0 (0.6)	14.9 (0.6)	12.7 (0.4)	13.6 (0.5)
Median (IQR)	*0.0 (0.0-0.0)*	*0.0 (0.0-0.0)*	*0.0 (0.0-0.0)*	*0.0 (0.0-0.0)*	*0.0 (0.0-0.0)*	*0.0 (0.0-0.0)*	*0.0 (0.0-0.0)*	*0.0 (0.0-0.0)*	*0.0 (0.0-0.0)*	*0.0 (0.0-0.0)*	*0.0 (0.0-0.0)*
**RV** *Range: 0-160*											
Mean (SE)	42.6 (0.9)	41.1 (0.9)	35.4 (1.0)	34.1 (1.0)	32.1 (0.9)	31.9 (1.0)	32.3 (1.0)	30.8 (1.1)	29.4 (1.1)	26.4 (0.8)	23.7 (0.8)
Median (IQR)	*0.0 (0.0-50.7)*	*0.0 (0.0-39.3)*	*0.0 (0.0-36.5)*	*0.0 (0.0-36.5)*	*0.0 (0.0-36.4)*	*0.0 (0.0-36.3)*	*0.0 (0.0-36.4)*	*0.0 (0.0-36.2)*	*0.0 (0.0-36.1)*	*0.0 (0.0-26.0)*	*0.0 (0.0-9.5)*
**HepA** *Range: 0-32*											
Mean (SE)	9.9 (0.2)	9.4 (0.2)	8.9 (0.2)	8.1 (0.2)	7.6 (0.2)	7.3 (0.2)	7.3 (0.2)	6.8 (0.2)	6.2 (0.2)	5.8 (0.2)	5.8 (0.2)
Median (IQR)	*0.0 (0.0-31.1)*	*0.0 (0.0-31.1)*	*0.0 (0.0-31.0)*	*0.00 (0.0-15.1)*	*0.00 (0.0-6.4)*	*0.0 (0.0-2.8)*	*0.0 (0.0-3.3)*	*0.0 (0.0-0.0)*	*0.0 (0.0-0.0)*	*0.0 (0.0-0.0)*	*0.0 (0.0-0.0)*

DTaP, diphtheria-tetanus-acellular pertussis; HepA, hepatitis A; HepB, hepatitis B; Hib, *Haemophilus* influenzae type b; IPV, inactivated poliovirus; IQR, interquartile range; MMR, measles-mumps-rubella; PCV, pneumococcal conjugate vaccine; RV, rotavirus; SE, standard error; VAR, varicella

© 2024 Newcomer SR et al. *JAMA Network Open.*

eTable 4. Prevalence of Children Who Received All Vaccine Doses On-Time (i.e., Zero Days Undervaccinated), for the Combined 7-Vaccine Series and for Nine Recommended Vaccinations, Among US Children by Age 19 Months, National Immunization Survey-Child 2011-2021

	**NIS-Child Survey Year**										
**Vaccination (s)**	**2011<br>n=19,144**	**2012<br>n=16,687**	**2013<br>n=13,611**	**2014<br>n=14,893**	**2015<br>n=15,167**	**2016<br>n=14,988**	**2017<br>n=15,333**	**2018<br>n=15,657**	**2019<br>n=16,365**	**2020<br>n=19,342**	**2021<br>n=17,967**
	**Weighted %**<br>(95% CI)<br>*unweighted sample size*										
**Combined 7-vaccine series**	**22.5%**<br>(21.4%-23.6%)<br>*n=4,710*	**26.0%**<br>(24.7%-27.2%)<br>*n=4,641*	**27.6%**<br>(26.2%-29.0%)<br>*n=4,037*	**27.6%**<br>(26.1%-29.0%)<br>*n=4,503*	**28.6%**<br>(27.3%-29.9%)<br>*n=4,834*	**29.7%**<br>(28.3%-31.1%)<br>*n=4,866*	**29.1%**<br>(27.8%-30.5%)<br>*n=5,071*	**31.6%**<br>(30.0%-33.1%)<br>*n=5,544*	**34.4%**<br>(32.8%-36.0%)<br>*n=6,354*	**34.9%**<br>(33.6%-36.1%)<br>*n=7,701*	**35.6%**<br>(34.2%-37.0%)<br>*n=7,184*
HepB	**63.4%**<br>(62.1%-64.8%)<br>*n=12,253*	**65.8%**<br>(64.3%-67.2%)<br>*n=11,150*	**67.0%**<br>(65.4%-68.7%)<br>*n=9,339*	**68.5%**<br>(67.0%-70.1%)<br>*n=10,447*	**67.8%**<br>(66.3%-69.2%)<br>*n=10,615*	**67.1%**<br>(65.5%-68.7%)<br>*n=10,352*	**69.2%**<br>(67.6%-70.8%)<br>*n=10,857*	**70.5%**<br>(68.8%-72.2%)<br>*n=11,395*	**72.1%**<br>(70.5%-73.8%)<br>*n=12,180*	**74.4%**<br>(73.2%-75.6%)<br>*n=14,750*	**76.1%**<br>(74.8%-77.4%)<br>*n=14,090*
DTaP	**56.2%**<br>(54.9%-57.6%)<br>*n=11,458*	**55.8%**<br>(54.3%-57.3%)<br>*n=9,890*	**55.9%**<br>54.3%-57.6%<br>*n=8,231*	**57.4%**<br>(55.7%-59.0%)<br>*n=8,946*	**55.9%**<br>(54.4%-57.4%)<br>*n=9,286*	**56.3%**<br>(54.7%-57.9%)<br>*n= 9,236*	**56.2%**<br>(54.5%-57.8%)<br>*n=9,391*	**58.0%**<br>(56.2%-59.7%)<br>*n=9,810*	**59.8%**<br>(58.1%-61.5%)<br>*n=10,557*	**60.0%**<br>(58.7%-61.4%)<br>*n=12,683*	**60.8%**<br>(59.3%-62.3%)<br>*n=11,974*
Hib	**39.1%**<br>(37.8%-40.4%)<br>*n= 8,195*	**43.5%**<br>(42.0%-44.9%)<br>*n=7,752*	**44.8%**<br>(43.2%-46.3%)<br>*n=6,549*	**44.7%**<br>(43.1%-46.3%)<br>*n=7,153*	**43.6%**<br>(42.2%-45.1%)<br>*n=7,385*	**46.1%**<br>(44.5%-47.7%)<br>*n=7,543*	**44.0%**<br>(42.4%-45.5%)<br>*n=7,601*	**47.2%**<br>(45.4%-48.9%)<br>*n=8,098*	**48.3%**<br>(46.6%-50.0%)<br>*n=8,865*	**48.8%**<br>(47.4%-50.1%)<br>*n=10,635*	**49.0%**<br>(47.5%-50.4%)<br>*n=9,689*
PCV	**50.4%**<br>(49.0%-51.7%)<br>*n=10,514*	**50.4%**<br>(48.9%-51.9%)<br>*n=9,022*	**51.2%**<br>(49.6%-52.9%)<br>*n=7,583*	**51.8%**<br>(50.1%-53.4%)<br>*n=8,301*	**52.4%**<br>(50.9%-53.9%)<br>*n=8,679*	**52.6%**<br>(51.0%-54.2%)<br>*n=8,646*	**51.7%**<br>(50.0%-53.3%)<br>*n=8,710*	**53.4%**<br>(51.6%-55.2%)<br>*n=9,225*	**55.6%**<br>(53.9%-57.4%)<br>*n=9,969*	**56.2%**<br>(54.9%-57.6%)<br>*n=12,078*	**57.5%**<br>(56.0%-59.0%)<br>*n=11,408*

© 2024 Newcomer SR et al. *JAMA Network Open.*

Vaccination (s)	NIS-Child Survey Year										
	**2011 n=19,144**	**2012 n=16,687**	**2013 n=13,611**	**2014 n=14,893**	**2015 n=15,167**	**2016 n=14,988**	**2017 n=15,333**	**2018 n=15,657**	**2019 n=16,365**	**2020 n=19,342**	**2021 n=17,967**
	**Weighted %**<br>(95% CI)<br>*unweighted sample size*										
IPV	**74.8%**<br>(73.6%-76.0%)<br>*n=14,857*	**75.1%**<br>(73.8%-76.3%)<br>*n=12,938*	**74.8%**<br>(73.4%-76.3%)<br>*n=10,678*	**75.9%**<br>(74.4%-77.3%)<br>*n=11,622*	**74.2%**<br>(72.8%-75.5%)<br>*n=11,803*	**74.9%**<br>(73.5%-76.4%)<br>*n=11,775*	**75.0%**<br>(73.5%-76.5%)<br>*n=12,121*	**75.6%**<br>(73.9%-77.3%)<br>*n=12,469*	**76.6%**<br>(75.1%-78.2%)<br>*n=13,240*	**77.6%**<br>(76.4%-78.8%)<br>*n=15,950*	**80.1%**<br>(78.8%-81.4%)<br>*n=15,144*
MMR	**79.3%**<br>(78.2%-80.4%)<br>*n=15,246*	**78.3%**<br>(77.0%-79.5%)<br>*n=13,287*	**81.2%**<br>(79.9%-82.4%)<br>*n=11,004*	**79.6%**<br>(78.4%-80.9%)<br>*n=12,052*	**81.7%**<br>(80.5%-82.8%)<br>*n=12,576*	**81.9%**<br>(80.7%-83.2%)<br>*n=12,465*	**82.6%**<br>(81.3%-83.8%)<br>*n=12,819*	**81.9%**<br>(80.5%-83.2%)<br>*n=13,128*	**82.0%**<br>(80.6%-83.5%)<br>*n=13,851*	**85.7%**<br>(84.7%-86.6%)<br>*n=16,889*	**84.5%**<br>(83.4%-85.6%)<br>*n=15,753*
VAR	**78.6%**<br>(77.5%-79.7%)<br>*n=15,051*	**77.8%**<br>(76.6%-79.0%)<br>*n=13,113*	**81.1%**<br>(79.9%-82.3%)<br>*n=10,933*	**79.9%**<br>(78.6%-81.1%)<br>*n=11,956*	**80.6%**<br>(79.5%-81.8%)<br>*n=12,358*	**80.6%**<br>(79.3%-81.9%)<br>*n=12,136*	**80.3%**<br>(79.0%-81.6%)<br>*n=12,479*	**80.1%**<br>(78.7%-81.6%)<br>*n=12,871*	**80.8%**<br>(79.4%-82.3%)<br>*n=13,568*	**83.1%**<br>(82.1%-84.2%)<br>*n=16,392*	**82.4%**<br>(81.3%-83.6%)<br>*n=15,278*
RV	**58.5%**<br>(57.2%-59.9%)<br>*n=11,769*	**60.7%**<br>(59.3%-62.1%)<br>*n=10,500*	**63.9%**<br>(62.3%-65.5%)<br>*n=9,067*	**65.0%**<br>(63.3%-66.6%)<br>*n=10,166*	**65.7%**<br>(64.2%-67.1%)<br>*n=10,469*	**66.4%**<br>(64.9%-68.0%)<br>*n=10,498*	**65.1%**<br>(63.5%-66.8%)<br>*n=10,730*	**67.6%**<br>(65.9%-69.3%)<br>*n=11,166*	**69.4%**<br>(67.8%-71.1%)<br>*n=12,013*	**69.8%**<br>(68.5%-71.1%)<br>*n=14,358*	**71.8%**<br>(70.5%-73.2%)<br>*n=13,560*
HepA	**65.0%**<br>(63.7%-66.3%)<br>*n=12,247*	**66.9%**<br>(65.6%-68.3%)<br>*n=11,262*	**68.4%**<br>(66.9%-69.9%)<br>*n=9,481*	**71.2%**<br>(69.7%-72.6%)<br>*n=10,666*	**73.0%**<br>(71.7%-74.3%)<br>*n=11,334*	**73.9%**<br>(72.5%-75.3%)<br>*n=11,174*	**73.9%**<br>(72.5%-75.3%)<br>*n=11,592*	**76.0%**<br>(74.5%-77.5%)<br>*n=12,170*	**77.8%**<br>(76.3%-79.2%)<br>*n=12,938*	**79.5%**<br>(78.4%-80.6%)<br>*n=15,747*	**79.4%**<br>(78.2%-80.6%)<br>*n=14,697*

CI, confidence interval; DTaP, diphtheria-tetanus-acellular pertussis; HepA, hepatitis A; HepB, hepatitis B; Hib, *Haemophilus* influenzae type b; IPV, inactivated poliovirus; MMR, measles-mumps-rubella; NIS-Child, National Immunization Survey-Child; PCV, pneumococcal conjugate vaccine; RV, rotavirus; VAR, varicella

© 2024 Newcomer SR et al. *JAMA Network Open.*

eTable 5. Associations Between Socioeconomic, Demographic, Household and Other Factors and On-Time Receipt of the Combined 7-Vaccine Series, National Immunization Survey-Child 2011-2021

	Total study sample N = 179,154	Outcome: On-time vaccination (i.e., zero days undervaccinated) n = 59,445 (29.7% of total study population)			
	No. (weighted %)	Unadjusted PR	(95% CI)	Adjusted[a] aPR	(95% CI)
**Survey Year**	--	1.041	(1.036-1.045)	1.034	(1.029-1.038)
**Poverty status**					
Above poverty, >$75,000 family income	74,479 (31.4%)	Reference		Reference	
Above poverty, ≤$75,000 family income	58,961 (32.4%)	0.742	(0.719-0.765)	0.905	(0.874-0.937)
Below poverty	39,564 (30.2%)	0.574	(0.552-0.596)	0.838	(0.792-0.886)
Missing	6,150 (6.0%)	N/A		N/A	
**Insurance coverage**					
Private insurance only	96,284 (43.1%)	Reference		Reference	
Any Medicaid[b]	61,461 (44.0%)	0.679	(0.659-0.700)	0.876	(0.839-0.914)
Other insurance[c]	9,073 (4.6%)	0.733	(0.687-0.783)	0.865	(0.809-0.925)
Uninsured	8,229 (5.5%)	0.522	(0.479-0.568)	0.688	(0.627-0.755)
Missing	4,107 (2.9%)	N/A		N/A	
**Sex**					
Female	86,906 (48.8%)	Reference		Reference	
Male	92,248 (51.2%)	0.991	(0.964-1.019)	0.983	(0.956-1.010)
**Race and ethnicity[d]**					
Non-Hispanic White only	104,908 (46.9%)	Reference		Reference	
Non-Hispanic Black only	14,986 (12.9%)	0.723	(0.687-0.760)	0.865	(0.820-0.913)
Non-Hispanic other and multiple races	23,864 (13.1%)	0.966	(0.926-1.008)	1.025	(0.983-1.070)
Hispanic	35,396 (27.1%)	0.871	(0.838-0.905)	1.070	(1.029-1.114)
**Census region**					
South	66,992 (38.9%)	Reference		Reference	
West	40,480 (24.4%)	0.941	(0.900-0.985)	0.888	(0.849-0.930)
Midwest	38,411 (20.8%)	1.008	(0.979-1.038)	0.960	(0.931-0.989)
Northeast	33,271 (15.9%)	0.900	(0.870-0.931)	0.833	(0.805-0.863)
**Geographic mobility**					
Has moved across state lines since birth	18,791 (10.0%)	0.672	(0.636-0.710)	0.681	(0.644-0.720)
Has not moved across state lines since birth	160,363 (90.0%)	Reference		Reference	
**Number of vaccine providers**					

© 2024 Newcomer SR et al. *JAMA Network Open.*

Zero	2,426 (0.9%)	N/A		N/A	
One	143,375 (81.2%)	Reference		Reference	
Two	29,948 (16.1%)	1.060	(1.021-1.100)	1.090	(1.039-1.143)
Three or more	3,405 (1.9%)	0.960	(0.865-1.066)	1.070	(0.959-1.193)
**Vaccine provider facility type**					
All private facilities	98,820 (56.3%)	Reference		Reference	
All public facilities	18,804 (12.2%)	0.741	(0.702-0.781)	0.943	(0.891-0.999)
All hospital facilities	28,022 (13.3%)	0.985	(0.947-1.023)	1.038	(0.998-1.079)
All military/other facilities	4,531 (2.7%)	0.728	(0.648-0.817)	0.812	(0.723-0.911)
Mixed facility types	26,034 (14.3%)	0.966	(0.927-1.007)	1.026	(0.971-1.083)
Missing	2,943 (1.1%)	N/A		N/A	
**Maternal education**					
<12 years	17,222 (15.8%)	0.595	(0.564-0.627)	0.837	(0.784-0.894)
12 years	30,552 (26.0%)	0.682	(0.656-0.708)	0.867	(0.828-0.908)
>12 years	45,168 (22.5%)	0.746	(0.721-0.772)	0.897	(0.864-0.931)
College graduate	86,212 (35.8%)	Reference		Reference	
**Number of children <18 years in the household**					
One	48,163 (28.0%)	Reference		Reference	
Two or three	107,418 (57.9%)	0.830	(0.806-0.855)	0.831	(0.807-0.856)
Four or more	23,573 (14.2%)	0.590	(0.557-0.624)	0.663	(0.626-0.702)
**Home ownership**					
Home was owned or being bought	111,159 (50.8%)	Reference		Reference	
Home was rented	61,610 (45.1%)	0.708	(0.687-0.730)	0.884	(0.852-0.916)
Other arrangement	5,861 (3.7%)	0.668	(0.612-0.729)	0.850	(0.775-0.931)
Don't know or refused	524 (0.4%)	N/A		N/A	

aPR, adjusted prevalence ratio; CI, confidence interval; NIS-Child, National Immunization Survey-Child; No., number; PR, prevalence ratio

[a] Due to missing covariate data, 166,316/179,154 observations were included in multivariable analyses (90.3%, weighted), including 56,607/59,445 children with zero days undervaccinated and complete covariate data (92.4%, weighted).
[b] If the child was on any form of Medicaid, alone or in combination with other types of health insurance status, the child was classified as Any Medicaid in the NIS-Child survey.
[c] Other insurance includes CHIP, IHS, Military, Tricare, CHAMPUS, or CHAMPVA (alone or in combination with private insurance).
[d] These are the race and ethnicity categories provided in the NIS-Child public-use datasets. The non-Hispanic other and multiple races category includes Asian, American Indian, Alaska Native, Native Hawaiian, Pacific Islander, and/or multiple races.

© 2024 Newcomer SR et al. *JAMA Network Open.*

eTable 6. Associations Between Socioeconomic, Demographic, Household and Other Factors and On-Time Receipt of the Combined 7-Vaccine Series, Among a Subcohort of US Children Who Completed the Combined 7-Vaccine Series by Age 19 Months, National Immunization Survey-Child 2011-2021

	**Outcome: On-time vaccination (i.e., zero days undervaccinated)** n = 59,445 (29.7% of total study population) vs. **Up-to-date at age 19 months, with delays (i.e., ≥1 day undervaccinated)** n = 49,570 (27.7% of the total study population)			
	**Unadjusted**		**Adjusted[a]**	
	**PR**	**(95% CI)**	**aPR**	**(95% CI)**
**Survey year**	1.026	(1.023-1.030)	1.024	(1.020-1.028)
**Poverty status**				
Above poverty, >$75,000 family income	Reference		Reference	
Above poverty, ≤$75,000 family income	0.884	(0.861-0.908)	0.966	(0.937-0.995)
Below poverty	0.785	(0.760-0.811)	0.934	(0.889-0.980)
**Insurance coverage**				
Private insurance only	Reference		Reference	
Any Medicaid[b]	0.838	(0.817-0.860)	0.922	(0.888-0.956)
Other insurance[c]	0.866	(0.819-0.916)	0.915	(0.864-0.968)
Uninsured	0.820	(0.766-0.877)	0.906	(0.840-0.976)
**Sex**				
Female	Reference		Reference	
Male	1.002	(0.979-1.026)	1.001	(0.978-1.025)
**Race and ethnicity[d]**				
Non-Hispanic White only	Reference		Reference	
Non-Hispanic Black only	0.861	(0.826-0.898)	0.931	(0.890-0.974)
Non-Hispanic other and multiple races	0.995	(0.961-1.031)	1.023	(0.987-1.061)
Hispanic	0.943	(0.914-0.974)	1.039	(1.004-1.075)
**Census region**				
South	Reference		Reference	
West	0.968	(0.932-1.006)	0.938	(0.903-0.974)
Midwest	1.001	(0.977-1.026)	0.967	(0.943-0.992)
Northeast	0.901	(0.876-0.927)	0.869	(0.844-0.895)

© 2024 Newcomer SR et al. *JAMA Network Open.*

**Geographic mobility**				
Has moved across state lines since birth	0.866	(0.826-0.907)	0.861	(0.820-0.903)
Has not moved across state lines since birth	Reference		Reference	
**Number of vaccine providers**				
One	Reference		Reference	
Two	0.998	(0.967-1.030)	0.999	(0.959-1.041)
Three or more	0.978	(0.899-1.063)	0.990	(0.903-1.086)
**Vaccine provider facility type**				
All private facilities	Reference		Reference	
All public facilities	0.897	(0.856-0.939)	0.993	(0.944-1.045)
All hospital facilities	1.047	(1.016-1.079)	1.071	(1.038-1.104)
All military/other facilities	0.878	(0.795-0.969)	0.897	(0.811-0.993)
Mixed facility types	1.003	(0.969-1.038)	1.054	(1.007-1.103)
**Maternal education**				
<12 years	0.801	(0.766-0.838)	0.919	(0.869-0.973)
12 years	0.861	(0.834-0.889)	0.950	(0.913-0.987)
>12 years	0.886	(0.861-0.912)	0.958	(0.927-0.990)
College graduate	Reference		Reference	
**Number of children <18 years in the household**				
One	Reference		Reference	
Two or three	0.911	(0.888-0.934)	0.909	(0.886-0.932)
Four or more	0.812	(0.775-0.851)	0.842	(0.803-0.883)
**Home ownership**				
Home was owned or being bought	Reference		Reference	
Home was rented	0.860	(0.838-0.883)	0.946	(0.917-0.976)
Other arrangement	0.816	(0.755-0.882)	0.895	(0.825-0.972)

aPR, adjusted prevalence ratio; CI, confidence interval; NIS-Child, National Immunization Survey-Child; PR, prevalence ratio

[a] Due to missing covariate data, 103,436/109,015 observations were included in multivariable analyses (92.1%, weighted). The following variables were missing data: vaccine provider facility type (n=264 missing observations, 0.2%, weighted), poverty status (n=3,493 missing observations, 5.6%, weighted), home ownership (n=294 missing observations, 0.4%, weighted), and insurance coverage (n=1,739 missing observations, 2.1%, weighted).

[b] If the child was on any form of Medicaid, alone or in combination with other types of health insurance status, the child was classified as Any Medicaid in the NIS-Child survey.

[c] Other insurance includes CHIP, IHS, Military, Tricare, CHAMPUS, or CHAMPVA (alone or in combination with private insurance).

[d] These are the race and ethnicity categories provided in the NIS-Child public-use datasets. The non-Hispanic other and multiple races category includes Asian, American Indian, Alaska Native, Native Hawaiian, Pacific Islander, and/or multiple races.

© 2024 Newcomer SR et al. *JAMA Network Open.*

eTable 7. Prevalence of Children Who Received All Vaccine Doses On-Time (i.e., Zero Days Undervaccinated), for the Combined 7-Vaccine Series by Age 19 Months, by Poverty Status and Health Insurance Status, National Immunization Survey-Child 2011 and 2021

	**NIS-Child 2011 survey year**	**NIS-Child 2021 survey year**	**Percentage point difference in on-time vaccination from 2011 survey to 2021 survey**
	**Weighted % (95% CI)**	**Weighted % (95% CI)**	**Percentage (95% CI)[a]**
Above poverty, >$75,000 family income	27.3% (25.4%-29.1%)	43.4% (41.3%-45.5%)	16.1 (13.3-18.8)
Above poverty, ≤$75,000 family income	25.1% (23.0%-27.2%)	35.0% (32.4%-37.6%)	9.9 (6.7-13.2)
Below poverty	17.3% (15.5%-19.0%)	24.4% (21.6%-27.2%)	7.1 (3.7-10.5)
Private insurance only	26.5% (25.0%-28.1%)	45.0% (43.0%-47.1%)	18.5 (16.0-21.1)
Any Medicaid[b]	20.7% (18.9%-22.5%)	27.4% (25.3%-29.4%)	6.7 (3.9-9.5)
Other insurance[c]	22.6% (15.8%-29.3%)	32.4% (27.4%-37.4%)	9.8 (1.6-18.1)
Uninsured	18.7% (13.8%-23.7%)	22.3% (14.6%-30.1%)	3.6 (-5.7-13.0)

CI, confidence interval; NIS-Child, National Immunization Survey-Child

[a] Bootstrap 95% normal-based CIs for these differences were constructed by resampling households 2,000 times within each year and stratum.
[b] If the child was on any form of Medicaid, alone or in combination with other types of health insurance status, the child was classified as Any Medicaid in the NIS-Child survey.
[c] Other insurance includes CHIP, IHS, Military, Tricare, CHAMPUS, or CHAMPVA (alone or in combination with private insurance).

© 2024 Newcomer SR et al. *JAMA Network Open.*

eTable 8. Associations Between Socioeconomic, Demographic, Household and Other Factors and On-Time Receipt of the Combined 7-Vaccine Series, Including Effect Modification by Poverty Status, National Immunization Survey-Child 2011-2021

	Outcome: On-time vaccination (i.e., zero days undervaccinated) n = 59,445 (29.7% of total study population)			
	Unadjusted		Adjusted[a]	
	PR	(95% CI)	aPR	(95% CI)
**Survey Year**	1.041	(1.036-1.045)	1.046	1.040-1.052
**Poverty status**				
Above poverty, >$75,000 family income	Reference			
Above poverty, ≤$75,000 family income	0.742	(0.719-0.765)	--	--
Below poverty	0.574	(0.552-0.596)	--	--
**Year x poverty status**				
Year, above poverty, >$75,000 family income			Reference	
Year, above poverty, ≤$75,000 family income	--	--	0.983	(0.974-0.993)
Year, below poverty	--	--	0.975	(0.964-0.987)
**Sex**				
Female	Reference		Reference	
Male	0.991	(0.964-1.019)	0.983	(0.956-1.010)
**Race and ethnicity[b]**				
Non-Hispanic White only	Reference		Reference	
Non-Hispanic Black only	0.723	(0.687-0.760)	0.864	(0.819-0.912)
Non-Hispanic other and multiple races	0.966	(0.926-1.008)	1.024	(0.982-1.069)
Hispanic	0.871	(0.838-0.905)	1.070	(1.028-1.113)
**Census region**				
South	Reference		Reference	
West	0.941	(0.900-0.985)	0.888	(0.849-0.930)
Midwest	1.008	(0.979-1.038)	0.960	(0.932-0.989)
Northeast	0.900	(0.870-0.931)	0.835	(0.807-0.864)
**Geographic mobility**				
Has moved across state lines since birth	0.672	(0.636-0.710)	0.682	(0.645-0.721)
Has not moved across state lines since birth	Reference		Reference	
**Number of vaccine providers**				
One	Reference		Reference	
Two	1.060	(1.021-1.100)	1.087	(1.037-1.142)

© 2024 Newcomer SR et al. *JAMA Network Open.*

Three or more	0.960	(0.865-1.066)	1.065	(0.955-1.191)
**Vaccine provider facility type**				
All private facilities	Reference		Reference	
All public facilities	0.741	(0.702-0.781)	0.943	(0.891-0.999)
All hospital facilities	0.985	(0.947-1.023)	1.037	(0.997-1.078)
All military/other facilities	0.728	(0.648-0.817)	0.812	(0.724-0.911)
Mixed facility types	0.966	(0.927-1.007)	1.026	(0.971-1.083)
**Maternal education**				
<12 years	0.595	(0.564-0.627)	0.833	(0.779-0.890)
12 years	0.682	(0.656-0.708)	0.866	(0.827-0.907)
>12 years	0.746	(0.721-0.772)	0.896	(0.864-0.931)
College graduate	Reference		Reference	
**Number of children <18 years in the household**				
One	Reference		Reference	
Two or three	0.830	(0.806-0.855)	0.831	(0.807-0.857)
Four or more	0.590	(0.557-0.624)	0.663	(0.626-0.702)
**Home ownership**				
Home was owned or being bought	Reference		Reference	
Home was rented	0.708	(0.687-0.730)	0.884	(0.852-0.916)
Other arrangement	0.668	(0.612-0.729)	0.851	(0.775-0.932)
**Insurance coverage**				
Private insurance only	Reference		Reference	
Any Medicaid[c]	0.679	(0.659-0.700)	0.878	(0.841-0.917)
Other insurance[d]	0.733	(0.687-0.783)	0.864	(0.807-0.923)
Uninsured	0.522	(0.479-0.568)	0.682	(0.622-0.749)

aPR, adjusted prevalence ratio; CI, confidence interval; NIS-Child, National Immunization Survey-Child; PR, prevalence ratio

[a] Due to missing covariate data, 166,316/179,154 observations were included in multivariable analyses (90.3%, weighted), including 56,607/59,445 children with zero days undervaccinated and complete covariate data (92.4%, weighted). The following variables were missing data: vaccine provider facility type (n=2,943 missing observations, 1.1%, weighted), poverty status (n=6,150 missing observations, 6.0%, weighted), home ownership (n=524 missing observations, 0.4%, weighted), and insurance coverage (n=4,107 missing observations, 2.9%, weighted).
[b] These are the race and ethnicity categories provided in the NIS-Child public-use datasets. The non-Hispanic other and multiple races category includes Asian, American Indian, Alaska Native, Native Hawaiian, Pacific Islander, and/or multiple races.
[c] If the child was on any form of Medicaid, alone or in combination with other types of health insurance status, the child was classified as Any Medicaid in the NIS-Child survey.
[d] Other insurance includes CHIP, IHS, Military, Tricare, CHAMPUS, or CHAMPVA (alone or in combination with private insurance).

© 2024 Newcomer SR et al. *JAMA Network Open.*

eTable 9. Associations Between Socioeconomic, Demographic, Household and Other Factors and On-Time Receipt of the Combined 7-Vaccine Series, Including Effect Modification by Health Insurance Status, National Immunization Survey-Child 2011-2021

	Outcome: On-time vaccination (i.e., zero days undervaccinated) n = 59,445 (29.7% of total study population)			
	Unadjusted		Adjusted[a]	
	PR	(95% CI)	aPR	(95% CI)
**Survey Year**	1.041	(1.036-1.045)	1.046	(1.040-1.051)
**Health insurance status**				
Private insurance only	Reference			
Any Medicaid[b]	0.679	(0.659-0.700)	--	--
Other insurance[c]	0.733	(0.687-0.783)	--	--
Uninsured	0.522	(0.479-0.568)	--	--
**Year x health insurance status**				
Year, private insurance only			Reference	
Year, any Medicaid[b]	--	--	0.971	(0.962-0.981)
Year, other insurance[c]	--	--	0.994	(0.970-1.018)
Year, uninsured	--	--	0.984	(0.955-1.014)
**Sex**				
Female	Reference		Reference	
Male	0.991	(0.964-1.019)	0.983	(0.956-1.010)
**Race and ethnicity[d]**				
Non-Hispanic White only	Reference		Reference	
Non-Hispanic Black only	0.723	(0.687-0.760)	0.863	(0.818-0.911)
Non-Hispanic other and multiple races	0.966	(0.926-1.008)	1.024	(0.982-1.069)
Hispanic	0.871	(0.838-0.905)	1.069	(1.028-1.112)
**Census region**				
South	Reference		Reference	
West	0.941	(0.900-0.985)	0.889	(0.850-0.930)
Midwest	1.008	(0.979-1.038)	0.959	(0.931-0.988)
Northeast	0.900	(0.870-0.931)	0.833	(0.805-0.862)
**Geographic mobility**				
Has moved across state lines since birth	0.672	(0.636-0.710)	0.682	(0.645-0.721)
Has not moved across state lines since birth	Reference		Reference	
**Number of vaccine providers**				
One	Reference		Reference	

© 2024 Newcomer SR et al. *JAMA Network Open.*

Two	1.060	(1.021-1.100)	1.087	(1.037-1.142)
Three or more	0.960	(0.865-1.066)	1.066	(0.954-1.190)
**Vaccine provider facility type**				
All private facilities	Reference		Reference	
All public facilities	0.741	(0.702-0.781)	0.943	(0.890-0.998)
All hospital facilities	0.985	(0.947-1.023)	1.037	(0.997-1.078)
All military/other facilities	0.728	(0.648-0.817)	0.815	(0.726-0.914)
Mixed facility types	0.966	(0.927-1.007)	1.026	(0.971-1.083)
**Maternal education**				
<12 years	0.595	(0.564-0.627)	0.832	(0.779-0.890)
12 years	0.682	(0.656-0.708)	0.868	(0.830-0.910)
>12 years	0.746	(0.721-0.772)	0.897	(0.865-0.932)
College graduate	Reference		Reference	
**Number of children <18 years in the household**				
One	Reference		Reference	
Two or three	0.830	(0.806-0.855)	0.832	(0.808-0.860)
Four or more	0.590	(0.557-0.624)	0.665	(0.628-0.706)
**Home ownership**				
Home was owned or being bought	Reference		Reference	
Home was rented	0.708	(0.687-0.730)	0.883	(0.851-0.915)
Other arrangement	0.668	(0.612-0.729)	0.851	(0.776-0.932)
**Poverty status**				
Above poverty, >$75,000 family income	Reference		Reference	
Above poverty, ≤$75,000 family income	0.742	(0.72-0.77)	0.913	(0.881-0.945)
Below poverty	0.574	(0.55-0.60)	0.836	(0.790-0.885)

aPR, adjusted prevalence ratio; CI, confidence interval; NIS-Child, National Immunization Survey-Child; PR, prevalence ratio

[a] Due to missing covariate data, 166,316/179,154 observations were included in multivariable analyses (90.3%, weighted), including 56,607/59,445 children with zero days undervaccinated and complete covariate data (92.4%, weighted). The following variables were missing data: vaccine provider facility type (n=2,943 missing observations, 1.1%, weighted), poverty status (n=6,150 missing observations, 6.0%, weighted), home ownership (n=524 missing observations, 0.4%, weighted), and insurance coverage (n=4,107 missing observations, 2.9%, weighted).
[b] If the child was on any form of Medicaid, alone or in combination with other types of health insurance status, the child was classified as Any Medicaid in the NIS-Child survey.
[c] Other insurance includes CHIP, IHS, Military, Tricare, CHAMPUS, or CHAMPVA (alone or in combination with private insurance).
[d] These are the race and ethnicity categories provided in the NIS-Child public-use datasets. The non-Hispanic other and multiple races category includes Asian, American Indian, Alaska Native, Native Hawaiian, Pacific Islander, and/or multiple races.

© 2024 Newcomer SR et al. *JAMA Network Open.*

eReferences

1. Opel DJ, Taylor JA, Mangione-Smith R, et al. Validity and reliability of a survey to identify vaccine-hesitant parents. *Vaccine*. 2011;29(38):6598-6605
2. Luman ET, Barker LE, Shaw KM, et al. Timeliness of childhood vaccinations in the United States: days undervaccinated and number of vaccines delayed. *JAMA*. Mar 9 2005;293(10):1204-11. doi:10.1001/jama.293.10.1204
3. Glanz JM, Newcomer SR, Narwaney KJ, et al. A population-based cohort study of undervaccination in 8 managed care organizations across the United States. *JAMA Pediatr*. Mar 1 2013;167(3):274-81. doi:10.1001/jamapediatrics.2013.502
4. Kurosky SK, Davis KL, Krishnarajah G. Completion and compliance of childhood vaccinations in the United States. *Vaccine*. 2016;34(3):387-394.
5. Newcomer SR, Freeman RE, Wehner BK, et al. Timeliness of early childhood vaccinations and undervaccination patterns in Montana. *Am J Prev Med*. Jul 2021;61(1):e21-e29. doi:10.1016/j.amepre.2021.01.038
6. Freeman RE, Thaker J, Daley MF, et al. Vaccine timeliness and prevalence of undervaccination patterns in children ages 0-19 months, U.S., National Immunization Survey-Child 2017. *Vaccine*. Jan 31 2022;40(5):765-773. doi:10.1016/j.vaccine.2021.12.037
7. Newcomer SR, Glanz JM, Daley MF. Beyond vaccination coverage: population-based measurement of early childhood immunization schedule adherence. *Academic Pediatrics*. 2022/08/20/ 2022;doi:https://doi.org/10.1016/j.acap.2022.08.003
8. Advisory Committee on Immunization Practices. *Recommended Child and Adolescent Immunization Schedule for age 18 years or younger*. 2022. Accessed September 1, 2022, https://www.cdc.gov/vaccines/schedules/downloads/child/0-18yrs-child-combined-schedule.pdf
9. GlaxoSmithKline. Package Insert - PEDIARIX. Accessed September 1, 2022, https://www.fda.gov/media/79830/download
10. Pasteur S. Package Insert - PENTACEL. Accessed September 1, 2022, https://www.fda.gov/media/74385/download
11. Advisory Committee on Immunization Practices. *Prior immunization schedules*. Accessed September 1, 2022. https://www.cdc.gov/vaccines/schedules/hcp/schedule-related-resources.html

© 2024 Newcomer SR et al. *JAMA Network Open.*
