# Supplementary material for: Vaccination Timeliness Among US Children Aged 0-19 Months, National Immunization Survey–Child 2011-2021
Source: JAMA Netw Open. 2024 Apr 12;7(4):e246440. doi: 10.1001/jamanetworkopen.2024.6440 (PMC11015353; doi:10.1001/jamanetworkopen.2024.6440)
Supplement: Supplement 2. — Data Sharing Statement [file jamanetwopen-e246440-s002.pdf]

Data Sharing Statement

Newcomer. Vaccination Timeliness Among US Children Aged 0-19 Months, National Immunization Survey–Child 2011-2021. *JAMA Netw Open*. Published April 12, 2024. doi:10.1001/jamanetworkopen.2024.6440

Data
Data available: Yes
Data types: Deidentified participant data, Data dictionary
How to access data: The CDC makes public use NIS-Child data files available on their website: https://www.cdc.gov/vaccines/imz-managers/nis/data-tables.html. The citation for the public use data files used in this study is: U.S. Department of Health and Human Services (DHHS). National Center for Immunization and Respiratory Diseases. The 2011-2021 National Immunization Survey-Child, Atlanta, GA: Centers for Disease Control and Prevention, 2012-2022.
When available: With publication

Supporting Documents
Document types: None

Additional Information
Who can access the data: The CDC makes public use NIS-Child data files available on their website for any user.
Types of analyses: The public use NIS-Child data files are available for analyses of vaccination uptake among US children surveyed at ages 19-35 months.
Mechanisms of data availability: The public use NIS-Child data files are available on the the CDC's website.
